# Prioritizing a research agenda on built environments and physical activity: a twin panel Delphi consensus process with researchers and knowledge users

**DOI:** 10.1186/s12966-023-01533-y

**Published:** 2023-12-07

**Authors:** Stephanie A. Prince, Justin J. Lang, Margaret de Groh, Hannah Badland, Anthony Barnett, Lori Baugh Littlejohns, Nicholas C. Brandon, Gregory P. Butler, Géna Casu, Ester Cerin, Rachel C. Colley, Louise de Lannoy, Iryna Demchenko, Holly N. Ellingwood, Kelly R. Evenson, Guy Faulkner, Liraz Fridman, Christine M. Friedenreich, Daniel L. Fuller, Pamela Fuselli, Lora M. Giangregorio, Neeru Gupta, Adriano A. Hino, Clare Hume, Birgit Isernhagen, Bin Jalaludin, Jeroen Lakerveld, Richard Larouche, Stephenie C. Lemon, Constantinos A. Loucaides, Jay E. Maddock, Gavin R. McCormack, Aman Mehta, Karen Milton, Jorge Mota, Victor D. Ngo, Neville Owen, Adewale L. Oyeyemi, António L. Palmeira, Daniel G. Rainham, Ryan E. Rhodes, Nicola D. Ridgers, Inge Roosendaal, Dori E. Rosenberg, Jasper Schipperijn, Sandra J. Slater, Kate E. Storey, Mark S. Tremblay, Mark A. Tully, Leigh M. Vanderloo, Jenny Veitch, Christina Vietinghoff, Stephen Whiting, Meghan Winters, Linchuan Yang, Robert Geneau

**Affiliations:** 1 Centre for Surveillance and Applied Research, Public Health Agency of Canada, 785 Carling Avenue, Ottawa, K1A 0K9 Canada; 2 School of Epidemiology and Public Health, Faculty of Medicine, University of Ottawa, Ottawa, Canada; 3 Alliance for Research in Exercise, Nutrition and Activity (ARENA), University of South Australia, City East Campus, Adelaide, South Australia; 4 Social and Global Studies Centre, RMIT University, Melbourne, Australia; 5 Mary MacKillop Institute for Health Research, Australian Catholic University, Melbourne, Victoria Australia; 6 School of Kinesiology, University of British Columbia, Vancouver, British Columbia Canada; 7 Population and Public Health, BC Centre for Disease Control, Vancouver, British Columbia Canada; 8 Peel Public Health, Region of Peel, Ontario, Canada; 9 Association pour la santé publique du Québec (ASPQ), Montréal, Québec, Canada; 10 School of Public Health, The University of Hong Kong, Hong Kong, Hong Kong SAR China; 11 Health Analysis Division, Statistics Canada, Ottawa, Ontario Canada; 12 Outdoor Play Canada, Ottawa, Ontario Canada; 13 Children’s Hospital of Eastern Ontario Research Institute, Ottawa, Ontario Canada; 14 Department of Psychology, Carleton University, Ottawa, Ontario Canada; 15 Department of Epidemiology, Gillings School of Global Public Health, University of North Carolina at Chapel Hill, Chapel Hill, North Carolina, USA; 16 Department of Mechanical and Industrial Engineering, Faculty of Applied Science and Engineering, University of Toronto, Toronto, Ontario Canada; 17 Department of Cancer Epidemiology and Prevention Research, Cancer Care Alberta, Alberta Health Services, Alberta, Canada; 18 Department of Oncology, Cumming School of Medicine, University of Calgary, Calgary, Alberta Canada; 19 Department of Community Health Sciences, Cumming School of Medicine, University of Calgary, Calgary, Alberta Canada; 20 Department of Community Health and Epidemiology, College of Medicine, University of Saskatchewan, Saskatoon, Saskatchewan Canada; 21 Parachute, Toronto, Ontario Canada; 22 Department of Kinesiology and Health Sciences, University of Waterloo, Waterloo, Ontario Canada; 23 Schlegel-UW Research Institute for Aging, Waterloo, Ontario Canada; 24 Department of Sociology, University of New Brunswick, Fredericton, New Brunswick Canada; 25 Health Sciences Graduate Program, School of Medicine and Health Sciences, Pontifícia Universidade Católica do Paraná, Curitiba, Paraná, Brazil; 26 School of Public Health, University of Adelaide, Adelaide, Australia; 27 Ottawa Public Health, Ottawa, Ontario Canada; 28 School of Population Health, University of New South Wales, Sydney, Australia; 29 Department of Epidemiology and Data Science, Amsterdam University Medical Centers, Vrije Universiteit Amsterdam, Amsterdam, the Netherlands; 30 Health Behaviours and Chronic Diseases, Amsterdam Public Health, Amsterdam, the Netherlands; 31 Upstream Team, Amsterdam University Medical Centers, Vrije Universiteit Amsterdam, Amsterdam, the Netherlands; 32 Faculty of Health Sciences, University of Lethbridge, Lethbridge, Alberta Canada; 33 Prevention Research Center, UMass Chan Medical School, Worcester, Massachusetts USA; 34 Ministry of Education, Sport and Youth, Nicosia, Cyprus; 35 School of Public Health, Texas A&M University, College Station, Texas USA; 36 Faculty of Kinesiology, University of Calgary, Calgary, Alberta Canada; 37 School of Planning, Architecture, and Landscape, University of Calgary, Calgary, Alberta Canada; 38 Faculty of Sport Sciences, Waseda University, Tokorozawa, Japan; 39 Maroondah City Council, Victoria, Australia; 40 Norwich Medical School, University of East Anglia, Norwich, UK; 41 Research Center in Physical Activity, health and Leisure (CIAFEL)-Faculty of Sports-University of Porto (FADEUP) and Laboratory for Integrative and Translational Research in Population Health (ITR), Porto, Portugal; 42 Canadian Institute of Planners, Ottawa, Ontario Canada; 43 Swinburne University of Technology, Melbourne, Victoria Australia; 44 Baker Heart and Diabetes Institute, Melbourne, Victoria Australia; 45 College of Health Solutions, Arizona State University, Phoenix, Arizona USA; 46 CIDEFES, Universidade Lusófona, Campo Grande, Lisboa, Portugal; 47 Healthy Populations Institute, Dalhousie University, Halifax, Nova Scotia Canada; 48 School of Health and Human Performance, Dalhousie University, Halifax, Nova Scotia Canada; 49 School of Exercise Science, Physical and Health Education, University of Victoria, Victoria, British Columbia Canada; 50 Kaiser Permanente Washington Health Research Institute, Seattle, Washington, USA; 51 Department of Sports Science and Clinical Biomechanics, University of Southern Denmark, Odense, Denmark; 52 Bachelor of Science in Public Health Program, School of Pharmacy, Concordia University Wisconsin, Mequon, Wisconsin USA; 53 School of Public Health, University of Alberta, Edmonton, Alberta Canada; 54 Department of Pediatrics, University of Ottawa, Ottawa, Ontario Canada; 55 Department of Health Sciences, Carleton University, Ottawa, Ontario Canada; 56 School of Medicine, Ulster University, Londonberry, United Kingdom; 57 ParticipACTION, Toronto, Ontario Canada; 58 School of Occupational Therapy, Western University, London, Ontario Canada; 59 Institute for Physical Activity and Nutrition (IPAN), School of Exercise and Nutrition Sciences, Deakin University, Geelong, Victoria Australia; 60 Infrastructure Canada, Ottawa, Ontario Canada; 61 World Health Organization Regional Office for Europe, Copenhagen, Denmark; 62 Faculty of Health Sciences, Simon Fraser University, Burnaby, British Columbia Canada; 63 Department of Urban and Rural Planning, School of Architecture, Southwest Jiaotong University, Chengdu, China

**Keywords:** Built environment, Physical activity, Delphi, Knowledge gaps, Knowledge translation

## Abstract

**Background:**

The growth of urban dwelling populations globally has led to rapid increases of research and policy initiatives addressing associations between the built environment and physical activity (PA). Given this rapid proliferation, it is important to identify priority areas and research questions for moving the field forward. The objective of this study was to identify and compare research priorities on the built environment and PA among researchers and knowledge users (e.g., policy makers, practitioners).

**Methods:**

Between September 2022 and April 2023, a three-round, modified Delphi survey was conducted among two independent panels of international researchers (*n* = 38) and knowledge users (*n* = 23) to identify similarities and differences in perceived research priorities on the built environment and PA and generate twin ‘top 10’ lists of the most important research needs.

**Results:**

From a broad range of self-identified issues, both panels ranked in common the most pressing research priorities including stronger study designs such as natural experiments, research that examines inequalities and inequities, establishing the cost effectiveness of interventions, safety and injuries related to engagement in active transportation (AT), and considerations for climate change and climate adaptation. Additional priorities identified by researchers included: implementation science, research that incorporates Indigenous perspectives, land-use policies, built environments that support active aging, and participatory research. Additional priorities identified by knowledge users included: built environments and PA among people living with disabilities and a need for national data on trip chaining, multi-modal travel, and non-work or school-related AT.

**Conclusions:**

Five common research priorities between the two groups emerged, including (1) to better understand causality, (2) interactions with the natural environment, (3) economic evaluations, (4) social disparities, and (5) preventable AT-related injuries. The findings may help set directions for future research, interdisciplinary and intersectoral collaborations, and funding opportunities.

**Supplementary Information:**

The online version contains supplementary material available at 10.1186/s12966-023-01533-y.

## Introduction

Habitual physical activity (PA) improves health and well-being and helps to reduce the risk for injury, many chronic conditions and premature mortality [1, 2]. However, prevailing inactive lifestyles mean inadequate numbers of children and adults meet national and international PA guidelines for health benefits [3]. There is growing recognition that ecological models can contribute to enhancing understanding of the facilitators and barriers to PA, notably as related to the influence of various aspects of the built environments on individual and social behaviours [4]. Built environments reflect the design and layout of the communities in which people live, work, learn and play and include land use for buildings and grounds, road and transit infrastructure, and parks and recreation facilities. In their review of the literature, Sallis et al. framed that the built environment exerts influence on PA behaviours in four key life domains: leisure/recreation, work/education, transportation, and household [4]. While the need for multiple levels of built environment interventions and policies related to PA are widely acknowledged, continuing challenges include identification of the optimal combination of study designs, target groups, built environment attributes, and policy processes to elevate understanding of which environmental changes will be most beneficial for PA promotion within and across populations [3].

The last two decades have seen a proliferation in the number of published studies investigating features of the built environment related to PA [5–8]. This is likely attributed to numerous factors including increased policy attention, availability of population- and place-based data sources, and targeted research funding opportunities examining obesogenic environments and rapidly growing urban settlements. Urban habitats continue to grow globally with 7 out of 10 people expecting to live in cities by 2050 [9]. In many high-income countries such as Canada, the ratio of urban-to-rural population ratio has already reached 80:20 [10]. Such shifting demographics along with social lifestyle changes have become the subject of many studies examining both broad and specific features of the built environment and PA [11–26].

Some members of the authorship group previously conducted a series of overviews of reviews to understand the current state of the evidence for built environments and PA across the life course [27, 28]. Evidence from the overviews suggest there is moderate-to-high certainty of positive associations between environments that support active transportation (AT; e.g., sidewalks, paths) and transport-related PA among youth and adults [27, 28]. Among youth, high certainty evidence suggests positive associations between play streets (i.e., closing a street to traffic) and PA, and between schoolyard design and PA [27]. Additionally, among adults, point of decision prompts such as signs to take the stairs, lead to increases in total PA [28]. The overviews identified gaps in the systematic review evidence such as a lack of research on preschool aged children, occupational PA, and the need for a life course perspective. Additional priorities included location-based approaches (e.g., studies that combine GPS with accelerometers) to capture the built environment where PA is taking place, and research to establish whether the built environment causally affects PA including evaluation of interventions and natural experiments [27, 28]. However, these overview-identified priorities do not necessarily represent all available evidence given the exponential growth of the literature in more recent years. Additionally, priorities identified within review evidence may not necessarily represent the most important priority areas for moving the field forward or for the advancement of policy and intervention design and implementation as they are most often generated by researchers rather than knowledge users (e.g., policy makers, practitioners).

To inform collaborative research between researchers and knowledge users, there is a need to identify shared research priorities to direct activities and practice. The Delphi method is a practical and structured means to obtain professional knowledge to derive a consensus from among a group of experts and informed respondents [29, 30] and has previously been used to identify research priority areas related to PA [31–33]. Participant anonymity is retained throughout the whole process and participants receive information in subsequent rounds on ratings from all respondents. A Delphi method can help to identify important areas for future research and to establish research priorities within the broader research and knowledge user communities.

The objective of this study was to identify and compare the ‘top 10’ research priorities on the built environment and PA from the perspectives of both researchers and knowledge users. Specifically, the study aimed to answer the question: what are the most important priorities requiring further study on human-made or modified aspects of the physical environments and their impacts on PA? The results are expected to help inform future research and further research priority setting activities; in particular, it represents valuable information for program and research funding organizations.

## Methods

### Study design and recruitment

A three-round, twin-panel modified Delphi survey method [30] was conducted among two independent groups (virtual panels) of researchers and knowledge users. The study targeted Canadian and international participants working in the field of the built environment and PA. Researchers were defined as individuals who carry out research activities related to the built environment and PA as their primary occupation [34]. Knowledge users were defined as individuals whose work is related to health policies, programs and/or practices and regularly apply or use research findings in their work [34]. Knowledge users could include, but were not limited to, practitioners including urban planners, policy makers, educators, decision makers, health care administrators, community leaders, or individuals working in public organizations. Ultimately, while specific individuals or organizations were identified as researchers or knowledge users, respondents were asked to self-identify during the survey as a researcher or knowledge user based on the above definitions.

The two panels were identified using active purposive sampling. Researchers were identified using four sources: 1) first authors from the individual reviews published in the last 10 years that were included in the built environment and PA overviews of reviews [27, 28, 35]; 2) first authors with five or more papers cited in the systematic reviews included in the overview of reviews [27, 28, 35]; 3) top cited authors (h-index > 15) identified in a Scopus ‘Researcher Discovery’ search for “built environment and physical activity”; and, 4) researchers whose projects were related to PA and the built environment and were funded by the Canadian Institutes of Health Research (CIHR) Healthy Cities Research Initiative [36]. Knowledge users were identified using four sources: 1) Canadian Federal agencies; 2) non-Federal Canadian agencies/organizations; 3) non-Canadian agencies/organizations; and 4) researcher suggested agencies/organizations obtained through round 1 of the researcher survey. International organizations working in these areas were identified using a grey literature scan. A conscious effort was made to identify a diverse group of respondents who conduct or use research on underserved areas or issues such as rural and urban design, women, minorities, those with a disability, and seniors. While these areas were targeted, those invited did not always agree to participate.

### Survey procedure

The modified Delphi method included three rounds of web-based surveys. Participants were contacted by email and invited to participate in the survey process with a direct web link to the survey. Communication and surveys were available in both English and French and the Round 1 survey was piloted in a group of knowledge user and researcher volunteers (*n* = 4) who did not participate in the study to ascertain time to completion and clarity. No changes were needed based on feedback. Participants were provided 3 weeks to complete each round with weekly reminders. The researcher round 1 survey commenced September 2022 and once closed, the knowledge user round 1 survey commenced October 2022. All three rounds were completed between September 2022 and April 2023 with approximately 4 weeks between each round. Microsoft Forms was used to collect responses for round 1 and Qualtrics surveys were used for rounds 2 and 3. Only those who completed each round were invited to participate in subsequent rounds. Participants were not made aware of the other panel until the beginning of the third round.

The first round asked participants to provide background demographic information to identify who the respondents were and the representativeness of those included in the process. They were then asked to provide their expert input on what they believe were the five highest priority gap research areas for the built environment and PA using open-ended questions. The round 1 researcher survey also asked respondents to identify any important knowledge users who worked in built environments and PA; eight knowledge users identified by researchers agreed to participate. Supplementary data file 1 provides a copy of the round 1 survey. At the end of round 1, one author (SAP) undertook a content analysis [37] to identify unique gaps submitted by all participants. The identified gaps were verified by the second author (JJL). If disagreements or discrepancies occurred, consensus was achieved through discussion between the two authors. Where possible, the original suggested text was retained. Gap phrasing was not necessarily kept the same between the panels.

In round 2, participants were asked to rate and rank the level of importance for each gap area identified from their respective panel in round 1. The list appeared in random order. Respondents were invited to first rate the importance of each research gap area from 1 (low importance) to 5 (high importance) and then to select 10 gaps that they felt were the most important. Upon identifying the 10 gaps, respondents were asked to rank order these gaps based on importance (1 = most important, 10 = least important). Based on the average rating and ranking, a top 20 list for each group was generated and provided to respondents in the third round.

The third round provided respondents with a list of the top 20 research gaps identified by their respective panel in round 2 including their round 2 importance scores and ranking. The list was created based on the frequency of appearance in the top 10 lists in round 2, followed by the average importance rating scores, and finally the rankings within the top 10 lists. Respondents were first asked if they agreed with the list as it appeared, or if they wanted to reorder the list and/or suggest changes to the list including combining gaps that may be related. If respondents agreed, the rank of the items was maintained, if not, participants had the opportunity to reorder the list. The average ranking provided in the third round determined the final order of the list. In addition to ranking, gaps were eligible for revision (i.e., combined, text change) when at least 20% of respondents suggested the same/similar change. Neither group saw nor provided feedback on the other group’s gap priority areas.

### Data analysis

Demographic characteristics of the respondents in round 1 are presented using proportions. For each round, the quantitative data and content analysis (of the open-ended round 1 responses) are presented.

## Results

### Participant characteristics

**Fig. 1:**
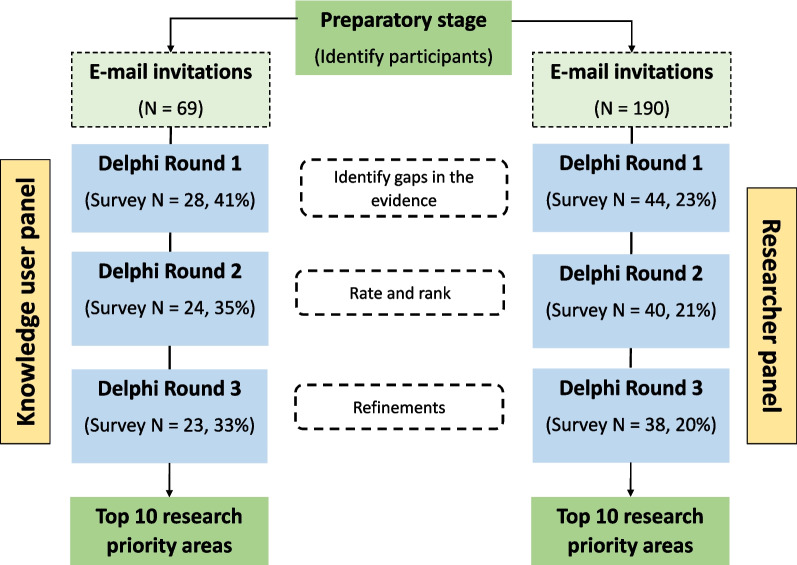
Study flow chart of participant engagement and retention across all three rounds of the twin-panelled (knowledge user panel and researcher panel) study

A total of 190 researchers and 69 knowledge users were invited to participate; of these 44 (23%) researchers and 28 (41%) knowledge users agreed to participate. Figure 1 shows the participant flow through the study. A total of 38 (20%) researchers and 23 (33%) knowledge users completed all three rounds. Table 1 describes the participant characteristics from round 1. Just over half of respondents in both the ‘researcher’ and ‘knowledge user’ groups identified as female. Most participants were from North America, with 43% of researchers and 71% of knowledge users from Canada. Among researchers, the majority (86%) were from academia, were employed in a traditional university academic appointment (84%), and had 10+ years of experience (75%). Researchers studied a variety of age groups, with preschool aged children being the least studied. Additionally, most researchers reported that cross-sectional studies were the study design most often used in their research, with randomized controlled trials, ecological studies and case-control studies being used the least. Among knowledge users, most were from some level of government (50%) or not-for-profit organizations (32%), 36% identified as also having a research role, always or frequently used research related to the built environment and PA in their work, and consulted a variety of sources for research evidence. Among both groups, their work focused on the transportation and recreation domains the most, with occupation being the least examined.

**Table 1:** Descriptive statistics for study panel participants during round 1

	Researchers (***n*** = 44)	Knowledge users (***n*** = 28)
**Female**	25 (57%)	15 (54%)
**Sector**		
Academia	38 (86%)	4 (14%)
Not-for-profit organization	1 (2%)	9 (32%)
Government (municipal, provincial/territorial, federal)	4 (9%)	14 (50%)
Non-academic institute	1 (2%)	0 (0%)
Consulting firm	0 (0%)	1 (4%)
**Position**		
University academic staff (e.g., researcher, lecturer, professor)	37 (84%)	5 (18%)
Researcher outside of academic setting (e.g., research institute, government)	6 (14%)	5 (18%)
Policy maker, planner, analyst, advisor	0 (0%)	9 (32%)
Public health practitioner	1 (2%)	5 (18%)
Urban planner, civil or transport engineer, architect	0 (0%)	6 (14%)
Research coordinator/manager, project lead	0 (0%)	3 (11%)
Trainee (e.g., graduate student, post-doctoral fellow)	1 (2%)	0 (0%)
Advocate	0 (0%)	1 (4%)
**Country or region of residence**		
Canada	19 (43%)	20 (71%)
Oceania	11 (25%)	3 (11%)
United States	7 (16%)	1 (4%)
South America	1 (2%)	0 (0%)
Europe	4 (9%)	4 (14%)
Africa	1 (2%)	0 (0%)
Asia	1 (2%)	0 (0%)
**Built environment and PA domain of focus**		
Transportation	29 (66%)	21 (75%)
Recreation	25 (57%)	23 (82%)
Occupation	3 (7%)	7 (25%)
School	11 (25%)	19 (68%)
All domains/total PA	22 (50%)	0 (0%)
**Years of experience in research**		
< 1–5 years	5 (11%)	–
6–10 years	6 (14%)	–
> 10 years	33 (75%)	–
**Age group focus of research (could select more than one option)**		
Preschool children (4 years and younger)	7 (16%)	–
Primary school-aged children (5–11 years)	16 (36%)	–
Youth/adolescents (12–17 years)	21 (48%)	–
Working-age adults (18–64 years)	26 (59%)	–
Older adults (65+ years)	20 (46%)	–
All ages (0+ years)	12 (27%)	–
**Study designs most often used in research (could select more than one option)**		
Cross-sectional studies	39 (89%)	–
Prospective cohort studies	26 (59%)	–
Qualitative studies	25 (57%)	–
Systematic reviews and meta-analyses	24 (55%)	–
Natural experiments	20 (46%)	–
Quasi-experimental studies (e.g., pre-post, non-controlled trials)	19 (43%)	–
Randomized controlled trials	10 (23%)	–
Ecological studies	9 (21%)	–
Case-control studies	3 (7%)	–
**Frequency of use of research related to built environment and PA**		
Always (approximately every day)	–	7 (25%)
Frequently (several times per month)	–	13 (30%)
Occasionally (about once per month)	–	4 (14%)
Rarely or never (less than once per month)	–	4 (14%)
**Sources of research evidence used (could select more than one option)**		
Peer-reviewed publications	**–**	25 (89%)
Organizational reports or newsletters	–	23 (82%)
Experts	–	23 (82%)
Conference presentations and/or abstracts	–	21 (75%)
Other (e.g., news media, professional associations, web-based data)	–	3 (11%)

*PA* physical activity

### Delphi results

During round 1, researchers submitted 144 unique responses that were grouped into 48 research priorities based on content (Supplementary Table 1). Knowledge users provided 88 unique responses that were grouped into 40 research priorities (Supplementary Table 2).

In round 2, the average rating (from 1 – least important to 5 – most important) ranged from 2.7 to 4.4 among researchers and 2.5 to 4.2 among knowledge users. The rating and ranking of the round 2 priorities are also presented in Supplementary Tables 3 and 4.

In round 3, among the researchers, after the re-ranking, the order changed slightly from round 2 with priorities 5 (i.e., Indigenous perspectives) and 6 (i.e., land-use policies), and 17 (i.e., residential relocation studies) and 18 (i.e., impacts in different social groups), swapping places. Nine researchers (24%) suggested that priorities 4 (i.e., inequities), and/or 11 (i.e., people living with disability) and/or 18 (i.e., different social groups) from round 2 be merged as they all thematically touched upon the need for research on inequalities and differences by social groups. Merging these three items did not change the order of the top 10 list. “Climate change perspectives” was removed from priority 5, as climate was addressed in item 3 and several researchers commented that these two topics should be separate. Among the knowledge users, after the re-ranking, the order of the top 10 remained unchanged. In the bottom ten there were slight changes with priorities 13 (i.e., speed limit reduction evaluation) and 14 (i.e., rural and non-urban communities) swapping, as well as priorities 16 (i.e., urban heat islands), 17 (i.e., leisure time PA) and 18 (i.e., 15-minute neighbourhoods) changing places. While several knowledge users suggested the need to merge items (e.g., priority #2 about different social groups and #6 about built environments among those living with disabilities), none of the suggestions were identified by at least 20% of the sample. The average ranking (1 = most important, 20 = least important) of the top 20 in round 3 are provided in Supplemental Tables 5 and 6.

Table 2 provides the final top 10 list for researchers and knowledge users. While the top 10 lists differed between the researcher and knowledge user groups, there were several areas of overlap. Similar priorities between the two groups were: stronger study designs including natural experiments (researcher priority #2, knowledge user priorities #5 + 7); inequalities and inequities (researcher priority #4, knowledge user priorities #1 + 2); considerations for climate change (researcher priority #3, knowledge user priority #4); cost effectiveness of interventions (researcher priority #9, knowledge user priority #3); and, safety and injuries (researcher priority #8, knowledge user priority #8). Additional priorities identified by researchers included: implementation science (priority #1); research that incorporates Indigenous perspectives (priority #5); land-use policies (priority #6); built environments that support active aging (priority #7); and, participatory research (priority #10). Additional priorities identified by knowledge users included: built environments and PA among people living with disabilities (priority #6); a need for national data on trip chaining, multi-modal travel, and non-work or school-related AT (priority #9); and, use of a systems thinking approach to AT (priority #10).

**Table 2:**
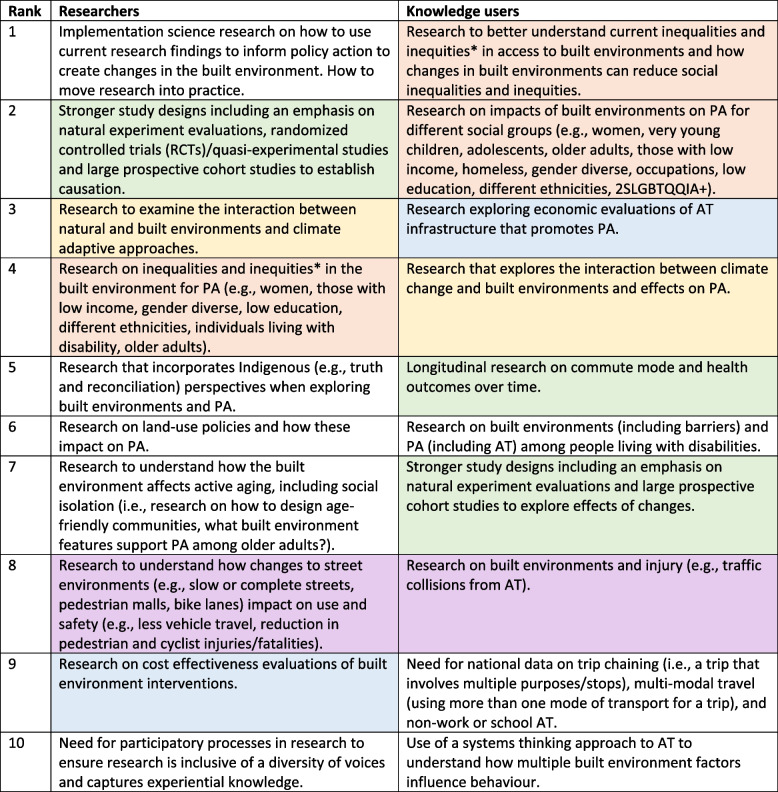
Ranked top 10 list of research priorities for the built environment and PA among researchers and knowledge users

Similar research priorities between researchers and knowledge users have been colour coded the same. ^a^Upon further review of supporting texts for these priorities, both inequality and inequity were often used. As a result, both terms were included in this final table

*AT* active transportation, *PA* physical activity

## Discussion

This study produced two lists of the top 10 research priorities for the built environment and PA: one among researchers and one among knowledge users. The top 10 research priorities from both groups addressed a broad range of gaps from study design and methodology, population considerations and intervention evaluations, through to the need for research on specific features of the built environment such as land-use policies and AT infrastructure. The knowledge user priorities often emphasized a focus on AT, while the research priorities were less prescriptive in terms of the types of PA. Although the 10 priorities were identified as stand-alone issues, it is recognized that there is likely some interaction and overlap across them. The use of two panels provides an opportunity to compare the top priorities between researchers and knowledge users.

### Similar research priorities between researchers and knowledge users

#### Stronger study designs including natural experiments (researcher priority #2, knowledge user priorities #5 + 7)

Both the researchers and knowledge users identified the need for stronger study designs. While observational designs have helped inform the relationship between the built environment and PA, larger prospective studies and rigorous evaluations using natural experiments will help to better establish causality and improve research quality. Additionally, the knowledge user panel described the need for longitudinal research on commute mode and health outcomes over time. This continues to be a priority reported in the literature [11, 26–28, 38, 39]. Natural experiments provide a means of evaluating the health impacts of policies, programs and other interventions that are implemented but for which the implementation is out of the control of researchers [40, 41]. They are valuable for understanding what changes in the built environment can increase PA, especially when a randomized controlled trial would be difficult to near impossible to implement due to an inability to randomly allocate participants to receive a built environment intervention (especially large-scale). In response, more recent reviews [13, 42–44] have included an emphasis on the inclusion of these study designs providing causal evidence to support built environment changes to increase PA (e.g., new infrastructure for walking, cycling and public transit associated with an increase in total PA and transportation-related PA [13]). However, there is a continued need to overcome biases and limitations often associated with natural experiments (e.g., lack of random selection of participants, control for confounding, control sites, valid and reliable measurement of PA, and outcome reporting bias [40, 45–47]).

#### Inequalities and inequities (researcher priority #4, knowledge user priorities #1 + 2)

Both groups identified the importance for ongoing research on inequalities and inequities in the built environments for PA with an emphasis on understanding differences in access and effects amongst population sub-groups (e.g., different ethnicities, individuals living with disability). Inequalities refer to “…differences, variations and disparities” in PA and built environments whereas inequities refer to unfair systematic differences for different groups of the population that are avoidable [48]. Understanding which groups have unequal access to positive built environment features or for whom different features of the built environment enable greater PA is imperative for the development of policies and interventions to reduce inequalities and inequities. A recent overview of reviews of population-based PA promotion approaches found an inconsistent consideration of equity in the evaluation of environmental interventions [49]. Research suggests that the association between the built environment and PA can differ by gender [50, 51], age [52–54] and socioeconomic status [55–57], and that inequalities exist for access to built environments that support PA by socioeconomic status and ethnicity [4, 58–60]. However, exploration of effects by sub-populations remains limited [27, 28]. Other groups which are under-represented as a research focus on access and effects of built environments for PA include the unhoused population, new immigrants, individuals living with a disability [61], different occupations, and members of the 2SLGBTQQIA+ community (represents those who are two-spirit, lesbian, gay, bisexual, transgender, queer, questioning, intersex, asexual, and all other sexual orientations and genders).

### Considerations for climate change (researcher priority #3, knowledge user priority #4)

Both groups included research priorities related to the interaction between the natural and built environments and the importance of including considerations for climate change and climate adaptation. Climate change poses a challenge to being physically active with higher global temperatures, extreme weather events, and reduced air and water quality having negative effects on PA levels [62]. In some instances, climate change may extend the outdoor PA ‘season’ for northern countries like Canada and Europe [63]. The built and natural environments can affect the sensitivity, exposure and adaptive capacity of individuals as they engage in active living [64]. For example, urban trees have been shown to associate positively with PA and have the capacity to reduce ultraviolet radiation, air and surface temperatures, and air pollutants [65]. As the transportation sector is responsible for about 37% of global CO_2_ emissions [66], AT could be an important climate change mitigation strategy [67, 68]. Future work is needed to explore how environments can be designed to promote PA in a changing climate, especially for different subgroups (e.g., disadvantaged areas where climate risks are greater due to less green area and tree coverage).

### Cost effectiveness of interventions (researcher priority #9, knowledge user priority #3)

Both groups included priority related to establishing the cost effectiveness of built environment interventions. The knowledge user priority, however, was specific to AT infrastructure. The current global costs of physical inactivity are estimated at INT$48 billion per year due to the treatment of preventable non-communicable diseases [69]. Economic evaluations of interventions are important for the development of policies given the costs associated with the interventions may be offset by the subsequent cost savings due to the promotion of PA and reduction of disease (i.e., cost-benefit analyses). Several types of economic analyses are available including cost-effectiveness, cost-benefit, cost-consequence, and social return on investment analyses. These evaluations are essential for comprehensive decision making and are part of evidence-based public health to ensure the most cost-effective interventions are identified and implemented [70]. To date, economic evaluations have often focused on interventions to increase AT [71], and have often considered only reductions in all-cause mortality as benefits of AT interventions [72]. There remains a need for natural experiments of built environments to promote PA with detailed economic evaluations [42]. Further, economic evaluations of natural experiments must address equity concerns, and include broader public health benefits from environmental interventions [73].

### Safety and injuries (researcher priority #8, knowledge user priority #8)

Finally, both groups included priorities that discussed safety and injuries related to engagement in AT. A systematic review of parental barriers towards children and youth’s AT to school identified the built environment (e.g., street connectivity), traffic safety and distance as some of the main barriers [74]. There is ample evidence to suggest that the built environment and traffic safety interventions are important correlates of injury associated with AT. For example, a study across several Canadian cities found that neighbourhoods with a higher proportion of residential land use and speed bumps were associated with lower child pedestrian and cyclist motor vehicle collisions [75]. Separated and protected cycling infrastructure has also been shown to reduce collisions and injury associated with AT [76, 77]. Most of the evidence on built environment features associated with safety and AT is cross-sectional [78], and there continues to be a need for evaluation of the effect of built environment changes (e.g., street environments, walking and cycling infrastructure) not only on use and PA, but also on reduction in vehicle travel and pedestrian injury [79]. Vision Zero is a global initiative trying to address safety using a systems approach with a goal to have zero fatalities or serious injuries as a result of road traffic accidents [80]. Evaluation and monitoring of Vision Zero is greatly needed to assess integration and effectiveness and promote uptake.

### Research priority differences between researchers and knowledge users

#### Implementation science (researcher priority #1)

While there was a lot of synergy between the researcher and knowledge user panels, there were differences. The top priority identified by knowledge users was the need to better understand inequalities, whereas the top priority identified by researchers was the need for implementation science research to advance current research knowledge into action and create changes in the built environment – *how to move research into practice*. Implementation science is a means to understand what needs to occur for interventions established as effective in research to become implemented into standard practice including understanding the barriers and facilitators and the strategies to overcome these barriers and facilitators [81, 82]. In turn, understanding the ‘what’, ‘for whom’ and ‘how’ an intervention and its implementation works is essential for replication across settings [83, 84]. Indeed, others have called for further implementation science research to better understand intervention effectiveness in population subgroups and how to move intervention evidence into practice [85–88]. Implementation science also plays a role in reducing inequalities, by understanding for whom the intervention works and why [89]; as such while the terminology is different, it incorporates an overlapping principle from understanding inequities (as per knowledge users) [90]. Several guides and frameworks exist for the implementation and scale up of PA (or general) interventions in practice [91] including the PRACTIS guide (PRACTical planning for Implementation and Scale-up) [82], the Framework for Effective Implementation [92], the RE-AIM (reach, effectiveness, adoption, implementation, maintenance) framework [93], and the Consolidated Framework for Implementation Research (CFIR) [94, 95].

#### Indigenous perspectives (researcher priority #5)

Researchers identified the importance of research that incorporate Indigenous perspectives and recognizes truth and reconciliation when exploring built environments and PA. The United Nations Declaration on the Rights of Indigenous Peoples (UNDRIP) recognizes that “…respect for indigenous knowledge, cultures and traditional practices contributes to sustainable and equitable development and proper management of the environment” [96]. This priority is well aligned with the Truth and Reconciliation Commission of Canada’s Calls to Action including #19 which called upon the federal government “…in consultation with Aboriginal peoples, to establish measurable goals to identify and close the gaps in health outcomes between Aboriginal and non-Aboriginal communities…” and #89 which called upon the federal government to “…support reconciliation by ensuring that policies to promote PA as a fundamental element of health and well-being, reduce barriers to sports participation, increase the pursuit of excellence in sport, and build capacity in the Canadian sport system, are inclusive of Aboriginal peoples.” [97] In response to the Calls to Action, Canadian federal research granting agencies have committed to supporting new models for Indigenous research and research training. These strategies are guided by the following key principles: “self-determination (fostering the right for First Nations, Inuit and Métis peoples to set their own research priorities), decolonization of research (respecting Indigenous ways of knowing and supporting community-led research), accountability (strengthening accountability in respecting Indigenous ethics and protocols in research and identifying the benefits and impacts of research in Indigenous communities), and equitable access (facilitating and promoting equitable access and support for Indigenous students and researchers)” [98]. The First Nation’s principles of ownership, control, access, and possession (OCAP®) establish how First Nations’ data and information should be collected, protected, used, and shared [99]. Indigenous research must be aligned with the goals and values of Indigenous peoples [100]. Several ethical guidelines for Indigenous health research exist including Chapter 9 of the Tri-Council Policy Statement 2 [101].

### Land-use policies (researcher priority #6)

Researchers identified research on land-use policies and how these impact PA as an important priority. While it was presented as a distinct priority, elements could also be captured under the evaluation of natural experiments. Land-use policies are “legislative or regulatory action, statements of intent, or guides to action issued by governments or organizations” [102] that target urban design such as city-level directives on residential density, street design, park placement, public transit, etc. Most systematic review evidence on land-use policies is older and is often mixed in terms of effects on PA [102, 103]. Additionally, the term “policy” is often conflated with “intervention” [104]; while there appears to be a large body of evidence supporting infrastructure (e.g., sidewalks, mixed land use) to promote PA, less has been done to evaluate policies (e.g., city transportation plans) [102]. While land-use policies can have positive effects, they have also been implicated in global injury and chronic disease through increased “…traffic exposure, noise, air pollution, social isolation, low PA, and sedentary behaviours” [105]. There is a continued need to monitor and evaluate the health consequences of urban design and for research to inform future healthy urban design and transport policies [105].

### Built environment and active aging (researcher priority #7)

Researchers identified a need for research to understand how built environments affect active aging including the design of age-friendly communities and understanding the features of the built environment that support PA among older adults. This priority was also related to the knowledge user priority #2 interested in the impacts of built environments on PA among different social groups including older adults. Physical inactivity is high globally (27.5%), but is higher among older age groups [106]. Aging in place is an important initiative identified by the World Health Organization (WHO), recognizing the growth of urban populations that are 60 years and older. The WHO has created a guide for age-friendly cities which targets eight areas of urban living including features of the built environment such as outdoor spaces and buildings, transportation, and housing [107]. Most findings on built environments and PA among older adults are equivocal with very limited longitudinal and experimental evidence [28, 108–110].

### Participatory processes in research (researcher priority #10)

Researchers identified the need for participatory processes in research to ensure that research is inclusive of a diversity of voices and captures experiential knowledge. This priority complements the need to address inequalities, as well as the incorporation of Indigenous and specific group perspectives. Participatory research is created and carried out *with* and *by* those who would benefit and use the research rather than *on* them as ‘subjects’ [111]. Participatory research is a major value-add in research ensuring that it is meaningful and in scope of the population, that the context for implementation is considered, and that the results are interpreted appropriately [111]. While participatory research has been included in PA interventions, there are no known reviews to have explored its use and effectiveness in built environment and PA research.

### People living with disabilities (knowledge user priority #6)

Knowledge users identified the need for research on built environments and PA among people living with disabilities. While this priority was suggested by a few to fall under the research on impacts of built environments and PA in different groups, it remained separate. Qualitative evidence suggests that elements of the built environment (e.g., benches, lighting, stop light timing) and perceived safety may positively support neighbourhood walking among people with a disability, however, mixed results have been reported in quantitative studies [61]. Evidence for built environments that support people living with disabilities to access destinations has largely focused on those with visual impairments, navigating crosswalks, and the cognitive elements of navigation [112]. Future research is needed to understand the role of the built environment on PA amongst people with different types of disabilities using valid and reliable measures of the built environment specific to people with disabilities and evaluating interventions [61, 112].

### Trip chaining, multi-modal travel and non-commuting AT (knowledge user priority #9)

Knowledge users identified a need for national data on trip chaining (e.g., trips with multiple purposes/stops), multi-modal travel (e.g., use of more than one mode of transportation per trip), and non-work or school-related AT. The built environment (e.g., destinations, transit stops, population and intersection density) has been shown to be associated with trip chaining [113, 114] and multi-modal travel [115], though most evidence has explored associations with AT in general. National trip chaining and travel data is often obtained through national travel surveys that include a single-day trip diary with origin, destination, route, and mode features. While many countries and cities include these surveys [116–119], Canada does not currently have a national household travel survey to assess complete daily travel patterns. Additionally, until the 2021 Canadian Census [120], data on multi-modal travel to work was not available.

### Use of a systems thinking approach to AT (knowledge user priority #10)

Finally, knowledge users identified the need for research to use a systems thinking approach to AT to understand how multiple built and other environmental factors influence travel behaviour. Systems thinking lends itself well to the ecological model of active living [4] recognizing that PA and AT are affected by multiple levels of influence (e.g., individual, social, environmental, policy) and multiple factors within each level. Complex systems methods such as participatory system mapping address the need to engage diverse perspectives [121]. Often built environment factors related to AT are examined individually, outside of the interaction with social and individual factors, and often without consideration of feedback (i.e. greater infrastructure promotes more walking which provides support to improve walking infrastructure [122]). The application of systems approaches such as system mapping, network analysis and system modelling have been used in the field of PA [123], though less than more individualistic approaches, and offer a means to consider whole systems and “…enhance the integration of socio-ecological models” [121]. Future work will benefit from incorporating and merging complex systems research, policy, and practice perspectives [121].

### Comparisons to previous research agenda-setting work

Previous groups have used similar expert consultations and consensus methods to identify priorities in the field (and beyond). Brownson et al. reported on a research agenda for environmental and policy approaches for promoting PA in the U.S. in 2006. The agenda was similarly developed using input from researchers and practitioners as part of the Physical Activity Policy Research Network (PAPRN). Although this agenda was conducted in the U.S. and almost 20 years ago, it captured many similar top priority areas including population subgroups, economic evaluation, implementation of policies, and measurement/methodology which included natural experiments and surveillance strategies [124]. Similarly, Reis et al. presented a research agenda for promoting PA in Brazil through environmental and policy approaches developed with input from practitioners and researchers using concept mapping in 2010–11. Among the cluster priority areas included evaluation and impact of policies, and economic benefits [125]. Jia et al. report on a top 10 research priority list in spatial life course epidemiology developed during a workshop as part of an international symposium in 2018. Similar priorities to those identified in the present Delphi study included the use of complex systems (e.g., systems thinking), health equity, and stronger study designs (by way of understanding residential self-selection, and improved exposure assessment in prospective studies) [126]. Despite the rapid proliferation of research, many priorities previously identified by other groups related to the built environment and PA still exist today.

### Study limitations

While this study used purposive sampling to identify a broad range of participants in terms of fields of study/work and geographic representation, given the sampling frame, it is not surprising that most participants were from Canada (especially among the knowledge users) with limited representation from low- and middle-income countries. Future work would benefit from understanding the similar and unique priority areas in low-, and middle- income countries. Participant demographics did suggest that there was adequate representation across the domains of built environments and PA and population age. However, the sample of researchers and knowledge users are not necessarily representative of all those working in the field. Unfortunately, the demographic information of non-respondents is unavailable; therefore, it is not possible to know how they differed from the sample. Ultimately, while a top 10 list was generated for both groups, it is possible that with a different group of participants a different set of priorities might emerge. Finally, while the lists provide direction for future research, given the rapidity in which the field is evolving, new priorities are likely to emerge, and an update of this Delphi exercise will likely be warranted in 5 years.

## Conclusions

This study used a modified Delphi method to identify and compare the top research priorities for built environments and PA among researchers and knowledge users. Five common top priorities emerged including the need for research using stronger study designs to better understand causality (e.g., longitudinal studies, natural experiments), research considering the interaction of natural and built environments and climate change and adaptation, research on inequalities and inequities in built environments and PA, economic evaluations of interventions, and research on safety and injuries related to engagement in AT. Most Delphi participants were from high-income countries, future work would benefit from understanding the similar and unique priority areas in low-, and middle- income countries. These identified priorities may help to provide direction for future research, collaborations, and the development of future funding opportunities. By creating a focused research agenda, we hope to advance as a unified built environments and PA research field .

### Supplementary Information

**Additional file 1:** **Supplementary data 1. **Delphi Survey Round 1. Built environments and physical activity.

**Additional file 2:** **Table S1. **Research priorities areas in the built environment and physical activity and identified by researchers in round 1.

**Additional file 3:** **Table S2. **Research priorities in the built environments and physical activity identified by knowledge users in round 1.

**Additional file 4:** **Table S3. **Research priorities in the built environments and physical activity identified by researchers in round 1 and rating and ranking from round 2.

**Additional file 5:** **Table S4. **Research priorities in the built environments and physical activity identified by knowledge users in round 1 and rating and ranking from round 2.

**Additional file 6:** **Table S5. **Average ranking of top 20 list in round 3 among researchers reported in order provided to researchers in round 3.

**Additional file 7:** **Table S6. **Average ranking of top 20 list in round 3 among knowledge users reported in order provided to knowledge users in round 3.

## Data Availability

All data generated or analysed during this study are included in this published article and its supplementary information files.
