# Supplementary material for: Prioritizing a research agenda on built environments and physical activity: a twin panel Delphi consensus process with researchers and knowledge users
Source: Int J Behav Nutr Phys Act. 2023 Dec 7;20:144. doi: 10.1186/s12966-023-01533-y (PMC10704660; doi:10.1186/s12966-023-01533-y)
Supplement: Supplementary file 1 — Additional file 1: Supplementary data 1. Delphi Survey Round 1. Built environments and physical activity. [file 12966_2023_1533_MOESM1_ESM.pdf]

Delphi Survey Round 1

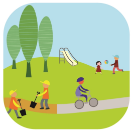

Built environments and physical activity

We appreciate you taking the time to complete this survey on research priorities for the built environment and its impact on physical activity. For the purpose of this survey, built environments are defined as physical environments that are human-made or human-modified and can include buildings and surrounding areas, community design, transportation infrastructure, and parks and recreation facilities. Built environments have the capacity to influence physical activity across four active living domains including: leisure/recreation; transportation; occupation/school; and, household.

* Required

Part A

To begin, we would like to ask you some questions to get a sense of who is responding to this survey.

1. We would like to contact you for the subsequent rounds of the survey, please include your email address below so that we may contact you again. Note: your email address will not be shared with others. *

2. What is your gender? Gender refers to current gender which may be different from sex assigned at birth and what is indicated on legal documents. Select all that apply. *

- [ ] Female
- [ ] Male
- [ ] Another gender (or please specify)
- [ ] Other

3. What is the primary sector you currently work in? *

- ○ Academia
- ○ Government - Municipal
- ○ Government - Provincial/Territorial
- ○ Government - Federal
- ○ Non academic institute
- ○ Not-for-profit organization
- ○ Independent consultant
- ○ Other

4. What best describes your professional position(s)? Select all that apply. *

- [ ] Trainee (e.g., graduate student or post-doctoral fellow)
- [ ] University-based academic staff (e.g., researcher, lecturer, professor)
- [ ] Researcher outside of academic setting (e.g. at a research institute, government)
- [ ] Healthcare practitioner (e.g., clinician)
- [ ] Public health practitioner
- [ ] Health policy maker, planner or analyst
- [ ] Urban planner
- [ ] Architect
- [ ] Other

5. What is your country of residence? *

- Afghanistan
- Albania
- Algeria
- Andorra
- Angola
- Antigua and Barbuda
- Argentina
- Armenia
- Australia
- Austria
- Azerbaijan
- The Bahamas
- Bahrain
- Bangladesh
- Barbados
- Belarus
- Belgium
- Belize

- ○ Benin
- ○ Bhutan
- ○ Bolivia
- ○ Bosnia and Herzegovina
- ○ Botswana
- ○ Brazil
- ○ Brunei
- ○ Bulgaria
- ○ Burkina Faso
- ○ Burundi
- ○ Cabo Verde
- ○ Cambodia
- ○ Cameroon
- ○ Canada
- ○ Central African Republic
- ○ Chad
- ○ Chile
- ○ China
- ○ Columbia
- ○ Comoros

- Congo, Democratic Republic of the
- Costa Rica
- Côte d'Ivoire
- Croatia
- Cuba
- Cyprus
- Czech Republic
- Denmark
- Djibouti
- Dominica
- Dominican Republic
- East Timor
- Ecuador
- Egypt
- El Salvador
- Equatorial Guinea
- Eritrea
- Estonia
- Eswatini
- Ethiopia

- [illegible]
- Fiji
- Finland
- France
- Gabon
- The Gambia
- Georgia
- Germany
- Ghana
- Greece
- Grenada
- Guatemala
- Guinea
- Guinea-Bissau
- Guyana
- Haiti
- Honduras
- Hungary
- Iceland
- India

- Indonesia
- Iran
- Iraq
- Ireland
- Israel
- Italy
- Jamaica
- Japan
- Jordan
- Kazakhstan
- Kenya
- Kiribati
- Korea, North
- Korea, South
- Kosovo
- Kuwait
- Kyrgyzstan
- Laos
- Latvia
- Lebanon

- Lesotho
- Liberia
- Libya
- Liechtenstein
- Lithuania
- Luxembourg
- Madagascar
- Malawi
- Malaysia
- Maldives
- Mali
- Malta
- Marshall Islands
- Mauritania
- Mauritius
- Mexico
- Micronesia, Federated States of
- Moldova
- Monaco

- Mongolia
- Montenegro
- Morocco
- Mozambique
- Myanmar
- Namibia
- Nauru
- Nepal
- Netherlands
- New Zealand
- Nicaragua
- Niger
- Nigeria
- North Macedonia
- Norway
- Oman
- Pakistan
- Palau
- Panama
- Papua New Guinea

- Paraguay
- Peru
- Philippines
- Poland
- Portugal
- Qatar
- Romania
- Russia
- Rwanda
- Saint Kitts and Nevis
- Saint Lucia
- Saint Vincent and the Grenadines
- Samoa
- San Marino
- Sao Tome and Principe
- Saudi Arabia
- Senegal
- Serbia
- Seychelles
- Sierra Leone

- [illegible]
- Singapore
- Slovakia
- Solomon Islands
- Somalia
- South Africa
- Spain
- Sri Lanka
- Sudan
- Sudan, South
- Suriname
- Sweden
- Switzerland
- Syria
- Taiwan
- Tajikistan
- Tanzania
- Thailand
- Togo
- Tonga

- ◯ Trinidad and Tobago
- ◯ Tunisia
- ◯ Turkey
- ◯ Turkmenistan
- ◯ Tuvalu
- ◯ Uganda
- ◯ Ukraine
- ◯ United Arab Emirates
- ◯ United Kingdom
- ◯ United States
- ◯ Uruguay
- ◯ Uzbekistan
- ◯ Vanuatu
- ◯ Vatican City
- ◯ Venezuela
- ◯ Vietnam
- ◯ Yemen
- ◯ Zambia
- ◯ Zimbabwe
- ◯ Other

6. If living in Canada, in what province or territory do you work?

- Alberta
- British Columbia
- Manitoba
- New Brunswick
- Newfoundland and Labrador
- Northwest Territories
- Nova Scotia
- Nunavut
- Ontario
- Prince Edward Island
- Québec
- Saskatchewan
- Yukon

7. Research/knowledge users are defined as individuals who regularly apply/use research findings to their work. Their work may be related to health policies, programs and/or practices. Research/knowledge users can include, but are not limited to practitioners including urban planners, policy makers, educators, decision makers, health care administrators, community leaders, or public organizations.

   Researchers are defined as individuals who carry out research activities as their primary occupation.

   Would you consider yourself a researcher or research/knowledge user? *

   ◯ Researcher

   ◯ Research/knowledge user

8. How many years of experience in researching the built environment **and** physical activity do you have? *

   ◯ Less than 1 year

   ◯ 1-5 years

   ◯ 6-10 years

   ◯ >10 years

9. What domains of the built environment and physical activity does your research focus on? Select all that apply. *

- [ ] Transportation
- [ ] Occupation
- [ ] School/education
- [ ] Recreation/leisure
- [ ] All domains/total physical activity
- [ ] Other

10. What age group does your research focus on? Select all that apply. *

- [ ] All ages (0+ years)
- [ ] Preschool children (4 years and younger)
- [ ] Primary school-aged children (5-11 years)
- [ ] Youth/adolescents (12-17 years)
- [ ] Working-age adults (18-65 years)
- [ ] Older adults (65+ years)
- [ ] Other

11. What methodologies do you use most often in your research? Select all that apply. *

- ☐ Cross-sectional studies
- ☐ Case-control studies
- ☐ Prospective cohort studies
- ☐ Randomized controlled trials
- ☐ Quasi-experimental studies (e.g., pre-post, non-controlled trials)
- ☐ Natural experiment evaluations
- ☐ Ecological studies
- ☐ Systematic reviews and meta-analyses
- ☐ Qualitative studies
- ☐ Other

12. We are conducting a separate Delphi survey with research/knowledge users. Research/knowledge users are defined as individual(s) who regularly apply/use research findings to their work related to built environments and physical activity. Their work may be related to health policies, programs and/or practices. Research/knowledge users can include, but are not limited to practitioners including urban planners, policy makers, educators, decision makers, health care administrators, community leaders, or public organizations.

    Please use the space below to provide information on an organization/agency and/or individual, including contact information if available, for any research/knowledge users you feel would be relevant to contact for this project.

    Example: John Doe (name), john.doe@web.ca (email address), JD Urban Health (organization)

13. How often do you use research findings related to the built environment **and** physical activity in your daily work? *

    - ◯ Always (approximately every day)
    - ◯ Frequently (several times per month)
    - ◯ Occasionally (about once per month)
    - ◯ Rarely (less than once per month)
    - ◯ Never (my work does not focus on the built environment and physical activity)

14. What domain(s) of the built environment/physical activity does your work involve? Select all that apply. *

- [ ] Transportation
- [ ] Occupation
- [ ] School/education
- [ ] Recreation/leisure
- [ ] Other

15. What are your sources of research evidence related to the built environment and physical activity? Select all that apply. *

- [ ] Peer-reviewed publications
- [ ] Conference presentations and/or abstracts
- [ ] Organizational newsletters
- [ ] Organizational reports
- [ ] Experts (e.g., discussion with researchers or others working in the field)
- [ ] Other

Part B

The next set of questions are seeking your input on what you see as the **priority research gap areas** on built environments and physical activity. These are open-ended questions to gather your expert knowledge on '***what we need to know***'.

Research gaps are areas that have not been answered appropriately, completely or at all. They may include lack of information on specific areas such as populations, research methods, built environment components, active living domains, or other important variables or conditions.

Please provide up to five (5) of the highest priority research gap areas/questions from your perspective. Provide additional information (including relevant citations) as it pertains to the rationale and evidence that supports its nomination as a high priority research area.

Examples:
Priority Research Area #1: There is a need for research examining changes in [built environment feature] on [physical activity domain] among [population].
Rationale: There are a lack of natural experiment evaluations of [built environment feature] on [physical activity domain] in [population]. Prince et al. *Health & Place* 2022
Priority Research Area #2: There is a need for longitudinal research using [type of measure] to assess [built environment feature and/or physical activity type].
Rationale: While cross-sectional studies have used [measure] to assess the association between [built environment] and [physical activity], no longitudinal studies have done so to assess causal associations. Prince et al. *Health & Place* 2022

16. **Priority Research Area #1** - Include any relevant rationale and evidence
*

17. **Priority Research Area #2** - Include any relevant rationale and evidence

18. **Priority Research Area #3** - Include any relevant rationale and evidence

19. **Priority Research Area #4** - Include any relevant rationale and evidence

20. **Priority Research Area #5** - Include any relevant rationale and evidence

This content is neither created nor endorsed by Microsoft. The data you submit will be sent to the form owner.

Microsoft Forms
