# Supplementary material for: Prioritizing a research agenda on built environments and physical activity: a twin panel Delphi consensus process with researchers and knowledge users
Source: Int J Behav Nutr Phys Act. 2023 Dec 7;20:144. doi: 10.1186/s12966-023-01533-y (PMC10704660; doi:10.1186/s12966-023-01533-y)
Supplement: Supplementary file 2 — Additional file 2: Table S1. Research priorities areas in the built environment and physical activity and identified by researchers in round 1. [file 12966_2023_1533_MOESM2_ESM.pdf]

Supplementary table 1. Research priorities areas in the built environment and physical activity and identified by researchers in round 1

| Priorities identified through from content analysis | Priority research areas submitted by researchers |
|---|---|
| Implementation science research on *how* to use current research findings to inform policy action to create changes in the built environment. How to move research into practice. | • There is a need for implementation science research on how to policy action toward built environment change happens.<br>• There is a need for implementation studies. i.e., how do we go from having the evidence that better bike lanes lead to more cycling to cities actually building more bike lakes. I.e., more studies on 'how do we make it happen' than on 'what works' or 'what effect does X have on health'.<br>• There is a need to take an implementation science approach to understand how interventions aimed at supporting health promoting built environments (i.e., designed to support PA) are designed, implemented, and maintained. There is a lack of research on the implementation of such interventions - including recent investments in our AT networks. There is a need to understand the essential conditions of implementation and maintenance of built environment interventions to understand how and why interventions are successful at increasing (or not) PA.<br>• Implementation research: I think there is well-established evidence that the built environment plays a role in shaping PA. There is now a need to better understand how we go about influencing policy and practice in this space to create supportive environments. How do we best advocate? Who do we work with? How do we convince policymakers/planners of the need to change/improve, particularly those outside the health sector who are responsible for much of our built environment?<br>• There is a difficulty in doing implementation science between programme implementers and evaluators. This kind of intersectoral work is rather rare and difficult to get funding for.<br>• We need more clear evidence about how to implement changes in the built environment that can affect the PA. Rationale: Considerable research has been conducted to understand how the built environment can affect the PA, including systematic reviews. We still need more evidence, but it is time to understand how can this evidence be implemented.<br>• Improved knowledge to action implementation re: built environment and the early years (PA levels)<br>• Effective policies and programs for shifting transport mode from car to active transport or a combination of active and public transport - all ages.<br>• Real life evaluation of programs, policies and initiatives is needed. Modelling is great, but dependent on the quality of data used and lots of assumptions. What happens in real life?<br>• Urban science to policy: how can science be better integrated in urban policy development? What structures/models can serve to better link the two separate worlds of academic research and urban planning/decision making? What are the science to urban policy models we can learn from?<br>• As the effects of built environment factors are often small at the population level, we also need to translate the findings that are meaningful to policy makers. For example, relating increase in PA to cardiovascular disease events prevented. Economic evaluation of health benefits (e.g., cost-benefit analysis) is also a very powerful tool and resonates with policy makers. A further benefit of modifying the built environment to increase PA (walking, cycling) is the relationship to climate change. Increased active travel, for example, means fewer cars on the roads and less tailpipe emissions leading to cleaner air as well as lower $CO_2$ emissions. These are the co-benefits of increasing active travel. How we measure this is complex and requires a transdisciplinary team of scientists.<br>• Research on the effects of improved AT infrastructure and policies on long-term active commuting are needed.<br>• There is a lack of research in the realm of implementation science related to the built environment. While a large body of evidence has focused on the WHAT (e.g., the association of built environment on health outcomes, effectiveness of built environment changes on improving health outcomes), there is almost no literature addressing the HOW (e.g., what strategies effectively accomplish built environment changes that influence positive health changes). |

| | |
|---|---|
| Stronger study designs including an emphasis on natural experiment evaluations, randomized controlled trials (RCTs)/quasi-experimental studies and large prospective cohort studies to establish causation. | • Across the literature, stronger study designs need to be employed including natural experiments, randomized trials if appropriate, and quasi-experimental designs that include a control group. Prospective cohort studies can also contribute, over and above the many cross-sectional studies that exist.<br>• Priority: There is a need for more residential relocation studies (longitudinal and quasi-longitudinal) and quasi-experiments to assess how changes in exposure to the neighbourhood built environment is associated with walking/PA among adults. Rationale: Much of the evidence that exists, and which currently informs urban and transportation policy is based on cross-sectional studies (from which causal inferences cannot be drawn).<br>• Natural experiments that include individual and social characteristics as moderators are needed. We have enough simple cross-sectional research and enough simple bivariate research. The nuances in terms of what works, for whom, under what conditions is the next stage in this research.<br>• There are a lack of natural experiment evaluations of changes to the built environment (green spaces, streets, schools). Particularly natural experiments focussing on particular demographic groups (e.g., older adults, teens, cultural groups) and including previous evidence on the refurbishment.<br>• There is a need for longitudinal or natural experimental study designs examining the association between supportive built environments and adolescent PA, while also accounting for different social environments (e.g., neighborhood crime/violence, social cohesion, etc.). Rationale: This relationship has been examined among adults, but little research has focused on adolescents.<br>• There is a need for more natural experiments investigating the effects of changes in the built environment on PA indicators. Rationale: natural experiments remain an underused approach in public health, and they have the potential to provide information about effectiveness of real-world interventions that are of particular interest for decision makers.<br>• There is a need for more longitudinal studies on the effects of the built environment on subsequent PA. Rationale: Cross-sectional studies represent the lion's share of previous research on the built environment, and they have important limitations, such as the inability to establish the direction of observed associations and demonstrate causality.<br>• Need for studies on the effect of environmental changes on changes in PA. Rationale example: One of the main weaknesses of the available evidence on physical environmental correlates of older adults' active transport is its cross-sectional nature. Cross-sectional studies cannot establish causality because they are affected by a range of threats to validity, residential self-selection being one of the most important even in good quality studies.<br>• Most evidence to date is based on cross-sectional research; more longitudinal research is needed to unravel causal relations between built environmental characteristics and PA.<br>• There is a need for more research capacity to conduct quasi-natural urban context experiments. This includes better funding of population cohorts and related continuous monitoring of participants, and of changing urban environments, both from a social and physical environment perspective.<br>• There is a need for research focusing on longitudinal relationships as cross-section studies can only give results that are less reliable.<br>• Natural experiments evaluation that could permit to understand better causality-related issues, impact and cost-effectiveness. Rationale: The majority of studies are cross-sectionals and can´t provide evidence of causality or cost-effectiveness<br>• More rigorous natural experiment evaluations are needed.<br>• Natural experiments and promotional experiments and their effect on perceived vs. actual environment change on PA. This will help to understand how the promotion of environments and the actual change in environments effect PA change.<br>• Prospective cohort studies that include diverse populations are needed that include measurements of the study population's built environments. Changes in activity levels along with changes in the built environment could be monitored over time, along with detailed covariate assessments to determine the impact of elements of the built environment on activity levels. |

| | |
|---|---|
| | • We need more longer-term prospective studies to establish causality. We need to take advantage of large cohorts with repeated measures that may have been established for other reasons to look at the effects of the built environment on PA. For example, one could then look at changes in PA patterns in cohort participants who change residential address i.e., moving from a low to a high walkability neighbourhood.<br>• Intervention research is required to provide evidence that factors in the built environment can be modified to improve health. Obviously, this is difficult to do in an established urban setting. Hence, we need to take advantage of natural experiments (e.g., establishment of a new cycling pathway) or greenfield developments (compare PA levels before and after moving to a greenfield development if the greenfield development is according to current concepts of a healthy urban neighbourhood).<br>• Methods based-research. The built environment field needs more innovation in study design and outcomes. Lack of strong designs and outcomes are a major barrier to conducting high quality research.<br>• There is a need for longitudinal/experimental research that examines what built environment factors may be associated with PA when accounting for gender. A recent review examined the existing literature, but the research included focused on two genders. |
| Research to examine the interaction between natural and built environments and climate adaptive approaches (e.g., climate adaptation). | • Understand the impact of the natural environment across a range of conditions and what can be done to alleviate (if needed) those conditions successfully to support PA. For example, I recently tried to help a country with very hot weather, and yet there was almost nothing to draw from in the literature.<br>• There is a need for longitudinal research exploring the mediating effect of climate risk conditions on the influence of built environment features on PA. Rationale: There dearth of prospective studies on how climatic conditions influence how built environment features impact PA.<br>• There is a need for research examining the challenges of building cycling infrastructure in northern climates. What are the best practices? How can cycling season be safely extended? Rationale: Cycling accidents are a growing concern in urban areas. How can Canada learn from other northern countries?<br>• There is a need for research examining the importance of greenness (and canopy cover, shade) at facilitating PA behaviour. Rationale: Warm summer temperatures make outdoor exercise difficult. Shade cover in dense urban areas is critically important to preserve but current development practices rarely take this into consideration.<br>• There is a need for research examining the extent /magnitude of urban green space needed in cities as climate changes, for health, equity et al reasons and mitigation against climate change (as air conditioning conversion not likely in many buildings). To what extent does the green asset become as important as built assets. |
| Research on inequities in the built environment for PA. | • In all areas of the built environment, consider how inequities may have been perpetuated and how they can be addressed going forward. Moreover, weigh both the positive and negative changes to the environment.<br>• There is a need to take an equity lens when researching the impact of built environments on PA.<br>• Are built-environment attributes protective, in the context of socio-economic differences in PA?<br>• Lack of studies examining factors contributing to disparities in PA: There are studies describing uneven distribution of PA, but we probably do not know much about how the built environment contributes to this.<br>• We need evidence on the equity effects of large PA interventions in general, including large community-based playground and park renovations. Rationale: Zenk et al. 2021 found that playground renovations in Chicago may have had unintended consequences, that resulted in increased neighborhood income and racial disparities in park use and park-based PA.<br>• Need for more work on urban health equity - how are environments distributed spatially and across social groups, and what are the consequences on health inequities? What are the urban policies that aim to address or redress these inequities: how are they developed, how are they implemented, who benefits?<br>• Need for large representative samples with a focus on equity that allow for subgroup and equity-based analysis. |

| | |
|---|---|
| | • Further research focusing on socio-economic inequities in access to safe and attractive/appealing green spaces for PA AND how this can be addressed both at a practical level (improving current green spaces and establishing new ones) but also at a system level (policies and strategic decisions around investment in green spaces in disadvantaged areas) is really important. |
| Research that incorporates Indigenous (e.g., truth and reconciliation) and climate change perspectives when exploring built environments and PA. | • There is a need for research that links truth and reconciliation, climate change, built environments and PA. |
| Research on land-use policies and how these impact on PA. | • Land use. In the realm of built environment and PA, a large body of research has examined transportation policies. There is much less research on land use policies and how they impact health behaviours and outcomes. |
| Research to understand how the built environment affects active aging, including social isolation (i.e., research on how to design age-friendly communities, what built environment features support PA among older adults). | • More research is needed to understand how the built environment affects active aging, including social isolation. This is important for the design of age-friendly communities.<br>• Can we learn from other countries that have built mini villages for elderly people that are bungalow-style and compact communities? With an emphasis on safe outdoor walking paths, greenness, social interactive, gardening etc.? This would need an ambitious partner with an eye on development/infrastructure. e.g., LandLab?? Rationale: We are facing a crisis in Canada with how to deal with an aging population. There is a drive for aging in place. How do we make vibrant, safe spaces for older Canadians to live in?<br>• There is a need for research examining changes in various built environment features among PA in older adults. |
| Research to understand how changes to street environments (e.g., slow or complete streets, pedestrian malls, bike lanes) impact on use and safety (e.g., less vehicle travel, reduction in pedestrian and cyclist injuries/fatalities). | • More research is needed to examine the association between slow or complete streets, or the creation of pedestrian malls and the reduction in pedestrian and bicyclist injuries/fatalities. Rationale: there really isn't anything known about how changes in street environments/adoption of slow street policies may make streets safer for all users.<br>• We need more and better evaluations of transportation interventions, such as bike lanes, bike share, etc. as to the impact not just on PA but also vehicle travel. |
| Research on cost effectiveness evaluations of built environment interventions. | • Economic research: There is a need to put an economic value on environmental changes in order to convince policymakers of the need for change. That is, if we create XX feature, what will be the direct benefit (health - e.g., lives saved, economic - e.g., $$ saved, environmental - e.g. carbon emissions reduced) to the community?<br>• Identifying health cost implications of different planning strategies: Behaviour-related or disease-related outcomes may not be convincing enough to planning decision makers.<br>• There is a need for more cost effectiveness evaluations of changes in the built environment.<br>• As with PA research in general, more economic evaluations are required |
| Need for participatory processes in research to ensure research is inclusive of a diversity of voices and captures experiential knowledge. | • Participatory processes: how can urban health science be more inclusive of a diversity of voices, and better capture experiential knowledge? Can we fund both conceptual and methodological developments along that line (e.g., along the lines of the 2009 Innovation in Health Research competition (https://www.researchnet-recherchenet.ca/rnr16/vwOpprtntyDtls.do?prog=796&view=browseArchive&browseArc=true&progType=CIHR-12&type=EXACT&resultCount=25&sort=deadline&next=1&all=1). |
| Research on features of the built environment that support PA among people living with disability. | • There is a need for more research examining built environment features associated with PA among people with disability. Rationale: for example, it has been shown that a focus on disability is clearly missing in the literature on AT and independent mobility.<br>• Appropriate neighbourhood scales and priority-built environment amenities for vulnerable populations (e.g., disability, culturally and linguistically diverse).<br>• There is a need for more inclusion of populations that experience disabilities in built environment research. Most research has focused on 'walkability' and 'bikeability', while ignoring the broader construct of 'active mobility' for people of all abilities. |

| | |
|---|---|
| Research to understand what policy levers are available to promote PA among urban populations. | • Would like to see more research on the policy mechanisms available or that work to support PA among urban populations (e.g., incentivizing behaviour change over the long term). |
| Research that applies a whole of systems approach to built environments and PA. | • Applying complexity and systems science to describe, assess and strengthen systems for population PA through action on the built environment. Whole of systems approaches are in their infancy and offer promising ways forward. |
| Research investigating how the built environment can enhance or inhibit the effectiveness of PA interventions. | • Priority: There is a need for more research investigating how the built environment can enhance or inhibit the effectiveness of PA interventions in adults. Rationale: PA interventions are implemented, often without consideration for how the built environment may support these interventions. Interventions that promote unstructured or self-directed PA may be the most likely to be impacted by the built environment.<br>• There is a need for researchers to combine individual level strategies for promoting PA with built environment changes (i.e.. multilevel approaches) in intervention studies. |
| Application of a life course perspective when examining built environment as a determinant of PA. | • Changes in built environment use over the life course.<br>• There is a need for research examining how urban densification is impacting people of different ages. Rationale: Published studies show a positive association between increased density and walkability with PA among working-age adults; however, evidence suggests the opposite is true for children and possibly older adults. Are we helping one group at the detriment of other groups? Children are arguably an important group to cater urban planning to given they have limited options to engage in PA.<br>• Evidence on the impact of changes to the environment on a range of PA behaviours - in all age groups. Natural experiment studies should include all ages, not just adults. There could be benefits for other age groups as well, but equally there could be unintended consequences. Important to know. |
| Research to examine how structural racism and discrimination contribute to limited PA opportunities/resources across marginalized communities. | • There is a need to examine how structural racism and discrimination contribute to limited PA opportunities/resources across marginalized communities, and how this influences PA levels. |
| Residential relocation studies to assess how changes in the built environment are associated with changes in PA. | • Priority: There is a need for more residential relocation studies (longitudinal and quasi-longitudinal) and quasi-experiments to assess how changes in exposure to the neighbourhood built environment is associated with walking/PA among adults. Rationale: Much of the evidence that exists, and which currently informs urban and transportation policy is based on cross-sectional studies (from which causal inferences cannot be drawn).<br>• I think there is a great need for self-selection type studies - where we aim to examine if people opt to live in an environment that is supportive of PA and active travel because they are already active or does moving to the environment change their behaviour. |
| Research on impacts of built environment on PA for different social groups (e.g., women, those with low income, gender diverse, low education, different ethnicities, older adults). | • There is a need to look at impacts of built environment change on PA for different social identities (e.g., women, people with low incomes, intersecting identities). Rationale: published evidence tends to present average effects. PA patterns by gender. Cities and built environments are experienced very differently across gender.<br>• There is a need to understand how neighbourhood features combined with other intersectional factors influence PA participation e.g., age, SES, ethnicity, education, gender. The populations that are under-represented in research are likely those most in need of supports to be physically active.<br>• Built environment initiatives often focus on the healthy adult e.g., take your bike! walk more! Older adults may have impairments that prevent them from being in physically active in those ways. They also may have more specific goals or needs e.g., falls prevention, muscle strengthening. Research on optimal ways to build in supports in the community to support adoption of movement guidelines in older adults, including balance and strength training guidelines.<br>• Need for large representative samples with a focus on equity that allow for subgroup and equity-based analysis. |

| | |
|---|---|
| | • Evidence on the impact of changes to the environment on a range of PA behaviours - in all age groups. Natural experiment studies should include all ages, not just adults. There could be benefits for other age groups as well, but equally there could be unintended consequences. Important to know.<br>• There is a need for research focusing on specific socio-demographic groups, such as elders, women, children, low-incomers, migrants, and the disabled.<br>• There is a need for studies on the effectiveness of built environment interventions and the impacts on PA ACROSS DIFFERENT CONTEXT, AND FOR DIFFERENT POPULATION GROUPS. Rationale: context matters, but we don't have comparative studies in different contexts (e.g., most in large metro areas, not in smaller towns, suburbs, etc.).<br>• There is a need to understand how socially and structurally vulnerable populations who do not have access to recreational facilities could use their physical surroundings for increased levels of PA. Most research has been done on higher SES populations who have a choice on where they do their PA.<br>• There are a lack of natural experiment evaluations of changes to the built environment (green spaces, streets, schools). Particularly natural experiments focussing on particular demographic groups (e.g., older adults, teens, cultural groups) and including previous evidence on the refurbishment.<br>• There is a need for research to examine the impact of greening spaces within the built environment on activity levels in different populations. For example, a recent review [Parrish et al., 2020, Sports Med] found few studies had evaluated such interventions in school-yard settings. |
| Research on built environments that support active outdoor free play in school-aged children. | • There is a lack of research on how to ensure that the built environment is conducive to school-aged children being able to play freely in their neighbourhoods, be independently mobile and engage in active transport.<br>• There is a need for research examining the features of the outdoor environment (natural and built) and relationships with active play among children and youth. Rationale: There is a pervasive assumption that built (and natural) environment features that are associated with greater levels of PA among adults are transferable to children - and this is fundamentally not the case - in fact, the desirable features may be in conflict.<br>• There is a need to study a broader base of features (the homage paid to mixed land use, population density and street connectivity is like a religion, but isolated and adult-centric thinking in my view) - think "playability" not just "walkability". And playability requires social and environmental features - and the environmental features of importance are probably more natural than built (e.g., is there open space, interstitial space, where kids could run, hide, build, explore - no "built" feature at all, but permission to use and play).<br>• There is a need for more research to identify built environment features that support PA in children. Rationale: previous research suggests that characteristics positively associated with PA in youth and adults (e.g., walkability), may be negatively associated with children's PA.<br>• Appropriate neighbourhood scales and priority built environment amenities for children.<br>• There is a need to explore youth perceptions of the built environment and how it relates to their PA - including active school travel and engagement with their environment. We often assume that we are creating spaces and places that are supportive of PA for those of all ages, but youth voice is very often lacking in research and practice. |
| Research to understand travel mode choice and influences on mode choice among different population groups (e.g., women, older adults, specific racial or ethnic groups). | • There is a need for examining travel mode choice and influences on mode choice among different population groups (e.g., women, older adults, specific racial or ethnic groups, etc.) |
| Research to examine the interaction between multiple environmental factors | • Studies on the built environment and PA need to better account for environmental confounders of associations. Rationale: Most studies in this research field have examined the effects of single or multiple environmental characteristics on PA without considering whether a characteristic might have lain on the causal pathway from another environmental characteristic to PA. |

| | |
|---|---|
| and mediators of the relationship between environments and PA. | • Need to take into account the inter-relationships between environment attributes in estimating their effects on PA. Rationale example: Neighbourhood residential density was related to seven other environmental attributes examined in Cerin et al. study, four of which acted as potential pathways of influence of densification on both transportation and recreation walking. These included street intersection, food and retail, recreation and public transport densities. All these attributes have been previously found to be positively related to either transportation, recreation or total walking in older adults.<br>• What are the necessary components of system change to cause behaviour change. Rationale: many studies fail to show an effect, but don't measure mediators of change or contextual factors that influence results.<br>• What factors mediate to relationships between built environment attributes and PA?<br>• What are the necessary components of system change to cause behaviour change? Rationale: many studies fail to show an effect, but don't measure mediators of change or contextual factors that influence results |
| Intervention research exploring how to influence AT to school in children. | • There is a need for programs addressing factors influencing children's active transport for the school journey. Few children walk and even fewer cycle for the school journey and while we have an understanding of what these influences are, there is a paucity of intervention studies addressing these factors.<br>• There is a need to understand the benefits of AT among children and youth. What strategies help to promote safe and AT to school? Rationale: The cumulative effect of small doses of daily PA has many health and social benefits. More research highlighting these benefits may help drive policies and investments in public health promotion campaigns to increase this. |
| Mixed methods research (e.g., qualitative and quantitative) to understand what is available and how it is perceived and barriers/facilitators of use. | • Research including mixed methods (qualitative and quantitative, perceived and objective methods, GIS and systematic observation). Rationale: The relationship of built environment and PA is complex and need to take into account a great amount of characteristics of the exposure that needs different approaches to be captured. For example, the GIS can provide a accurate measure of distances until parks, but might be need some measure of what is available in the park (obtained from systematic observation of the environment) or, even what the people perceive about the park to better understand how this kind of place can affect the PA behaviour. |
| Standardized methods for measuring elements in the built environment related to context-specific PA. | • There is a need to incorporate better and more standardized methods for measuring elements of the built environment to assess their individual and collective impact on PA levels. To date, these methods of characterizing the built environment have been minimally developed and there is considerable heterogeneity across studies on how these assessments and associations with PA have been made.<br>• We need to develop more sophisticated statistical methods to be able to account for the many built environment and individual risk factors that may impact on PA. Many built environment factors can be difficult to measure and then often only at the ecological level. Standardisation of such measures will assist researchers and also allow us to compare results across studies.<br>• There is a need for research into appropriate measures and study designs of the effect of the built environment on activity. Rationale: many studies fail to show an effect, because change is at a population/community level, which is hard to detect in small sample sizes.<br>• Priority: There is a need for more research that incorporates "context-specific" objective and self-report measures of PA that align with the contexts in which built environment variables are estimated (e.g., neighbourhood specific measures of PA). Rationale: Estimated effects of the built environment and PA, based on non-context specific measures of PA, are likely underestimated. |
| Longitudinal research combining GIS and GPS to device measures of PA to assess location of PA. | • Need for ongoing cohort studies to be able to conduct better quality natural experiment studies using accelerometer and GPS data.<br>• Research including mixed methods (qualitative and quantitative, perceived and objective methods, GIS and systematic observation). Rationale: The relationship of built environment and PA is complex and need to take into account a great amount of characteristics of the exposure that needs different approaches to be captured. For example, the GIS can provide a accurate measure of distances until parks, but might be need some measure of what is available in the park (obtained from systematic observation of the environment) or, even what the people perceive about the park to better understand how this kind of place can affect the PA behaviour.<br>• Specific measures of PA behaviour, including combination of Global Positioning System/accelerometer and questions that evaluate PA specifically conducted inside the neighborhood or area of exposure. Rationale: The PA is a complex behaviour, and the association between built environment characteristics and PA is very specific for the domain of PA (leisure time, commuting, labor, and household activities), |

| | |
|---|---|
| | type of PA (walking, cycling, sports, etc...) and place where the activity is conducted. So, better measures of PA are needed to better capture these relationships.<br>• There is a need for longitudinal research using GIS and GPS to measure the built environmental impact on PA behaviours of children and adolescents. Rationale: Cross-sectional studies using self-reported measures of the built environment have been inconsistently associated with PA behaviours of children and adolescents compared with adults. |
| Research integrating AT and health benefits in traffic models. | • integration of AT and its health benefits in traffic models. Many traffic models 'just' use travel time as an outcome, and do not consider long-term health outcomes, or pollution. |
| Development of a multi-sectoral and multidiscipline research framework for the built environment and PA. | • It would be useful to develop a research framework and intervention plan that supports perspectives from a variety of disciplines that can bring the expertise all together - public health, social justice, environment health/safety, planning, economics, rural, etc.<br>• There is a continued need to include urban design, planner, landscape architects in research on the built environment so that the physical infrastructure is correctly assessed along with the impact on human behaviour. Often multidisciplinary teams are not involved in research on the built environment which is a missed opportunity for better assessment and research.<br>• There is a need for a better understanding on how sectors can work together to create supportive environments for PA. For example (and this is one very specific example) - how can education, health, sport and recreation better work together to support active school travel?<br>• Increased integration of ECEs in the creation of PA-supportive spaces for the early years. |
| Need for modeling studies into co-benefits of built environment changes beyond PA. | • (modelling) studies into co-benefits of PA policy in the built environment. |
| Research on built environments in rural and small-town communities and other urban typologies. | • There is a need for research examining changes in neighbouring built environments on active transport and other leisure-time PA among residents of rural and small-town communities. Rationale: Much of the current research focuses on large urban areas.<br>• We continue to need to build the evidence on what built environment interventions are appropriate for facilitating PA in rural areas. Much research has been conducted and identified what works in urban areas, but evidence gaps for rural communities remain.<br>• There is a need for studies on the effectiveness of built environment interventions and the impacts on PA ACROSS DIFFERENT CONTEXT, AND FOR DIFFERENT POPULATION GROUPS. Rationale: context matters, but we don't have comparative studies in different contexts (e.g., most in large metro areas, not in smaller towns, suburbs, etc.).<br>• We have good evidence on the role of AT in support movement behaviours in urban areas. We need more studies (e.g. natural experiments, or RCTs) to identify the determinants of support for AT across a variety of urban typologies.<br>• There is a need for more evidence on modifiable built environment features on PA in rural settings. Policies and relevant systems, etc. are quite different in rural settings and much more research is needed. |
| Research on impact of built environments on PA in low- and middle- income countries. | a need for more research on built environment features that influence PA in children, youth, and adults from low- and middle-income countries.<br>e: most of the evidence stems from high-income countries.<br>a need for research examining changes in built environment features on leisure-time PA among the population in LMICs. Rationale: There is a lack of<br>experiment evaluation of built environment features on PA in the LMICs.<br>a need for research focusing on the Global South because the majority of previous studies have concentrated on the Global North. |
| Research examining the interaction between the social and built environments including social norms (e.g., active outdoor play, independent mobility). | • There is a need for research examining the intersection of social norms and social environment (e.g., acceptances, permissions, affordances, allowances, habits) and outdoor environment features/presence (natural and built) on independent mobility among children and youth. Rationale: The importance of built environment features is not universal, and likely less important than social acceptance of the freedom of children to roam their neighbourhoods safely and habitually, with a cushion of social capital. |

| | |
|---|---|
| | • There is a need for longitudinal or natural experimental study designs examining the association between supportive built environments and adolescent PA, while also accounting for different social environments (e.g., neighborhood crime/violence, social cohesion, etc.). Rationale: This relationship has been examined among adults, but little research has focused on adolescents.<br>• How do changes in the built environment affect social behaviours, which in turn influence individual behaviours? Rationale: I'm thinking here about herd effects, social contagion and the like (10.1038/ncomms14753) and how the built environment may be designed to change PA culture. For example, if a new bikeway is implemented, one should also think about how to promote it and engage people (especially the ones with more social influence) in its use; a built SOCIAL environment, instead of only a built PHYSICAL environment. |
| Better understanding of perceived vs. objective/actual environments and PA. | • Natural experiments and promotional experiments and their effect on perceived vs. actual environment change on PA. This will help to understand how the promotion of environments and the actual change in environments effect PA change. |
| Research on built environment interventions for PA across different contexts (e.g., within and outside neighbourhoods, specific locations). | • There is a need for studies on the effectiveness of built environment interventions and the impacts on PA ACROSS DIFFERENT CONTEXT, AND FOR DIFFERENT POPULATION GROUPS. Rationale: context matters, but we don't have comparative studies in different contexts (e.g., most in large metro areas, not in smaller towns, suburbs, etc.).<br>• There is a need for research examining how changes in different interdependent aspects of the neighborhood built environment impact on total and context-specific (i.e., within and outside the neighborhood) PA. Rationale: There is a lack of longitudinal and quasi-experimental studies in the field of environmental determinants of PA.<br>• There is a need for research focusing on the role of local or space-specific variables (e.g., teahouse in China) in shaping PA. Such variables may not be universal, but they have interesting connections with PA in some locations. These location-specific findings can also inform international academia.<br>• Linking interventions to promote movement to behavioural contexts. Majority of current intervention strategies are bereft of knowledge of context (places where movements occur and the timing of those movements). |
| Research that applies behaviour change theories to support behaviour change interventions. | • Application of theory and strategies from behavioural economics to support behavioural change interventions/experiments (e.g., carrot app). |
| Research to understand what components of the built environment are also associated with sedentary behaviour. | • There is a significant gap related to our understanding of the built and natural environment on sedentary time. A recent study we published found that higher greenness was associate with HIGHER sedentary time in older adults. Work is needed to better understand the interactions with PA too.<br>• Are the built environment attributes that are important for PA also important for sedentary behaviour?<br>• Car driving is sedentary behaviour and research is needed how transportation behaviours can be made more active (walking, cycling). This entails the drivability and walkability of neighbourhoods. |
| Development of better self-report measurement tools for travel behaviour that account for multi-modal trips or selecting different modes for different purposes or days (e.g., tools that move beyond "in a typical day how do you travel?"). | • There is a need for better self-report measurement tools for travel behaviour in surveillance measures. Things that can allow you to account for multi-modal trips or traveling via different modes on different days are needed. Too much national data is out there based on a terrible measurement tool (i.e., "on a typical day how do you travel to work?") that doesn't get at so many behaviours or factors. |
| Research to establish threshold values of environmental attributes associated with PA. | • Studies on the built environment and PA need to establish thresholds values of environmental attributes associated with meaningful PA targets. Rationale: Many cities worldwide do not have measurable policy targets that would facilitate the monitoring of progress on city planning interventions that influence health and health-related behaviours such as PA. This absence of city planning policy could partly be due to many countries not having measurable targets for reducing health risk factors. The absence of measurable city planning policy |

| | |
|---|---|
| | targets also stems from the dearth of clear guidance on thresholds of urban design and transport features needed to achieve the desired outcomes. To create healthy and sustainable cities, wherever possible, thresholds should be based on the empirical evidence of relationships of urban design and transport features with health-related behaviours and outcomes.<br>• Need to identify thresholds of environmental attributes associated with PA targets of public health significance. Rationale example: An essential characteristic of a healthy and sustainable city is a physically active population. Effective policies for healthy and sustainable cities require evidence-informed quantitative targets based on identified thresholds of built environment features associated with PA targets.<br>• There is a need for research examining the threshold of built environment features necessary for optimising leisure and transport-related PA. Rationale: There is a lack of research determining the threshold of built environment features on PA.<br>• There is a need to examine how changes in the built environment can be made that will increase the ease and availability of areas for PA amongst Canadians. This area of research is still relatively new and there are many gaps in knowledge about which elements of the built environment are most important to change to facilitate increased PA levels. |
| Research using international comparisons and a global lens. | • A global lens needs to be employed - not merely downtown Atlanta and Toronto. Employing truly representative samples, including low- and middle-income country samples, from both urban and rural areas, is needed. Rationale: Most data upon which guidance is based, is limited and biased to western, very-high-income countries and urban settings. |
| Research on built environment interventions in those at risk for poor mental health. | • There is a need for research examining changes in neighbouring built environments on active transport and other leisure-time PA among populations with or at risk of mental health problems. Rationale: Much of the current research focuses on physical health outcomes. |
| Research on impact of gentrification on PA. | • Impact of gentrification on health and wellbeing and any inequity trajectories. |
| Research to understand what environmental factors are related to different kinds of PA (e.g., bicycle use, walking, running, climbing, playing) compared to PA levels in general. | • What are the environmental factors related to different kinds of PA? For example, bicycle use, walking, etc.<br>• There is a need for research focusing on different types of PA, such as walking, running, cycling, and climbing mountains.<br>• More studies focusing on cycling. Rationale: Most of research testing the relationship between Built Environment and PA that has been conducted have not focused on the cycling, mainly commuting-related cycling. Considering that the relationship Built Environment-PA is behaviour specific, more studies focusing on cycling is needed. |
| Development of new analytical approaches (e.g., machine learning, latent class modeling, simulation models). | • Reliance on regression: new analytical approaches (e.g., machine learning) can be applied.<br>• We need to develop more sophisticated statistical methods to be able to account for the many built environment and individual risk factors that may impact on PA. Many built environment factors can be difficult to measure and then often only at the ecological level. Standardisation of such measures will assist researchers and also allow us to compare results across studies.<br>• Need to build tools that allow knowledge users to explore and simulate how built environment changes will change health including costing/benefit analyses. |
| Research to understand how different environments may affect the psychological response to PA and other movement-related behaviours. | • How different environments may affect PA and other movement-related behaviours and the psychological response to movement. Rationale: Again I'm thinking about interactions between environment, movement and another set of variables. In the previous one I was thinking on social variables, here I'm looking at psychological variables |
| Better understanding of to what extent are built environment relationships with overweight and obesity mediated by PA. | • What extent are the built environment relationships with overweight and obesity mediated by PA? |
| Research on built environments that support non-travel related PA. | • Priority: There is a need for more research that focusses on built environment variables that support PA other than transportation related walking and cycling in adults. Rationale: Built environment variables and indices that currently exist appear more strongly associated with |

| | |
|---|---|
| | transportation related PA (walking and cycling) than recreational PA. More evidence is needed about which built environment variables support recreational and leisure PA.<br>• Priority: There is a need for research that explores how the built environment supports walkability, cyclability, livability, vibrancy, and sustainability and how these dimensions support PA, social interaction, and health and wellbeing. Rationale: There are aspects of the built environment that can support PA, social interactions, and health and wellbeing that go beyond just walkability and cyclability. Healthy neighbourhoods are not just walkable but also support livability, vibrancy, and sustainability. A better understanding of the conceptual and operational definitions of these neighbourhood dimensions are needed. [McCormack et al. 2021. Cul-de-sacs make you fat': homebuyer and land developer perceptions of neighbourhood walkability, bikeability, livability, vibrancy, and health. |
| Research on e-microbilities (e.g., e-scooters, e-bikes) and their interaction with other modes of transportation and impacts on PA. | • Research on e-micromobilities and their interaction with other modes of transportation, as well as their impact on PA levels (light intensity) are needed. |
| Research using international comparisons of environmental attributes and PA. | • More international comparison: larger variabilities in exposure and outcome measures may help to identify key environmental attributes in promoting PA. |
| Research into other ways to restructure the environment to support PA in non-traditionally researched settings such as primary care and rehabilitation. | • There are other ways to restructure the environment to support PA. For example, thinking beyond outside or buildings but also settings - how can we best restructure approaches in primary care to promote PA? how can we promote rehabilitation or exercise post-hospital discharge, and build in community supports to encourage this? |

Note: some priorities submitted by respondents may appear in multiple content priorities. AT – active transportation, PA – physical activity
