# Supplementary material for: Prioritizing a research agenda on built environments and physical activity: a twin panel Delphi consensus process with researchers and knowledge users
Source: Int J Behav Nutr Phys Act. 2023 Dec 7;20:144. doi: 10.1186/s12966-023-01533-y (PMC10704660; doi:10.1186/s12966-023-01533-y)
Supplement: Supplementary file 3 — Additional file 3: Table S2. Research priorities in the built environments and physical activity identified by knowledge users in round 1. [file 12966_2023_1533_MOESM3_ESM.pdf]

Supplementary table 2. Research priorities in the built environments and physical activity identified by knowledge users in round 1

Research priorities identified through from content analysis	Research priorities submitted by knowledge users
Research to better understand current inequalities in access to built environments and how changes in built environments can reduce social inequalities.	• There is need to understand how in long run changes on built environment (such as parks and playgrounds) change health behaviours to tackle social and gender inequalities.<br>• Il est nécessaire de mener des recherches sur la disponibilité et l'accessibilité aux espaces verts au sein des quartiers plus défavorisés au Québec. Justification : Les parcs, les espaces publics et les terrains de jeu comptent parmi les lieux les plus fréquentés pour la pratique d'activités physiques. Toutefois, l'offre d'espaces verts publics n'est pas toujours répartie de façon équitable sur les territoires, notamment dans les quartiers plus défavorisés où les populations ont souvent un accès limité aux parcs et autres installations sportives ou de loisirs. Aussi, une attention particulière doit être portée aux enjeux d'accessibilité, en veillant à relier ces espaces aux différents milieux de vie via des infrastructures de transports actifs et collectifs et par un réseau de corridors verts favorables à la mobilité active.<br>• There is a need for research examining the availability and condition of sport and recreation infrastructure to support participation in both "organized" (e.g., registered sport activities) and "unorganized" (e.g., casual sport activities, cycling, etc.) sport and recreation. A further evaluation by socio-economic factors (e.g., which neighbourhoods have what infrastructure, and in what condition) would also be helpful. Rationale: There is limited available data on the state of sport and recreation infrastructure in general. The additional evaluation by socio-economic could help identify communities in greatest need of investment.<br>• The intersectionality of the built environment, PA and equity, diversity and inclusion is relatively new but essential in moving forward to impact those most vulnerable. Understanding how equity issues are related to the built environment and how that environment can facilitate or create barriers to PA will inform and influence future recommendations.
Research on impacts of built environments on PA for different social groups (e.g., women, very young children, adolescents, older adults, those with low income, homeless, gender diverse, occupations, low education, different ethnicities, 2SLGBTQQIA+)	• Research on design of public spaces and PA levels of adolescent girls.<br>• The relationship between social determinants of health, the built environment and PA. Rationale: There is a lack of information about the intersection between different social determinants of health (e.g., age, gender, race, occupation) and how they interact with factors related to the built environment and PA.<br>• There is a need for research examining changes in transport infrastructure on PA among all population groups.<br>• There is a need for research examining how the built environment can support people experiencing homelessness to engage in PA. Rationale: There is a great deal of research focused on homelessness related to food security, shelter, mental health, etc. but very little related to the importance of PA/sport/recreation for people experiencing homelessness and how they can be supported (in all aspects, including the built environment).<br>• There is a need for research examining changes in urban design on PA among older adults.<br>• Il est nécessaire de mener des recherches plus approfondies sur les saines habitudes de vies des de 0-5 ans, notamment sur l'activité physique (développement moteur) et les environnements favorisants cette activité. Il y a un manque de données permettant d'identifier les obstacles et les facilitants de l'APS qui ne permet pas d'appuyer les interventions sur des données probantes récentes. / There is a need for more research on healthy lifestyles for 0-5 year olds, including PA (motor development) and supportive environments. There is a lack of data to identify barriers and facilitators to PSA, which does not allow interventions to be based on recent evidence.<br>• Il est nécessaire de mener des recherches sur les environnements favorables par rapport à l'activité physique sportive pour la population issue de la communauté LGBTQ+. Il y a un manque d'évaluations complète sous forme d'expériences naturelles de l'effet aménagement de l'environnement bâti sur l'activité physique et sportive de cette communauté. / There is a need for research on the supportive environments for sport PA for the LGBTQ+ population. There is a lack of comprehensive evaluations in the form of natural experiments of the design effect of the built environment on PA and sport for this community.

	• There is a need for research examining changes in the quality of public realm on the level of PA across the various age groups. While the relationship between diversity, places of interest and safety was conclusive, the relationship b/w density on level of activity was inconclusive.
Research exploring economic evaluations of AT infrastructure that promotes PA.	• Economic arguments and analysis of AT infrastructure that promote PA, and its benefit to local business, which can support pushback from the community when road right-of-way is taken away from cars. Rationale: In Environmental Assessments this would be useful to support more AT. The communities will benefit from this and this needs to be communicated.<br>• More research on the impact on retail businesses of converting on-street car parking spaces to cycleways. It remains the major sticking point to getting better infrastructure in place in Australia.<br>• Meaningful economic impact analysis of AT infrastructure implementation. Both of the implementation itself (for example the cost savings for jurisdictions that integrate traffic calming, town squares and protected bike lanes as an essential service in all public works), as well as the effects on the public, specifically business (related to, for example, parking, or foot traffic or employee mental health, etc.).
Research that explores the interaction between climate change and built environments and effects on PA.	• The impact of climate change on the built environment and how it will influence PA. Rationale: The effects of climate change are being felt in many communities. It is unclear how factors like increased extreme weather events, heat episodes and other sequelae of climate change will impact PA dependent on the local built environment.<br>• Il est nécessaire de mener des recherches sur les changements de climatique par rapport à l'activité physique sportive pour la population et vise et versa. Il y a un manque d'évaluations complète sous forme d'expériences naturelles de l'effet des changements climatique sur l'activité physique et sportive pour la population et évaluer les liens de causalité. / There is a need for research on climate change in relation to population PA and vice versa. There is a lack of comprehensive evaluations in the form of natural experiments of the effect of climate change on PA and sport for the population and to evaluate the causal links.<br>• PA and AT has traditionally been investigated and explored in relation to the reduction of chronic diseases. However, we are increasingly realizing the intersectionality between PA and AT and climate change. Research needs to better integrate climate change and the co-benefits of reducing GHG emissions with other public health imperatives. This will enable more persuasive arguments and provide a more conclusive body of evidence that municipalities can use to advance sustainable AT and climate resiliency in the face of many other competing pressures.<br>• Greenhouse gas emissions reduction from AT. While different institutes (e.g., the California Air Resources Board) have attempted to quantify these benefits, most approximations rely on ungrounded assumptions and there does not appear to be a standardized methodology. While we've seen a couple stakeholder submissions that attempt to quantify these savings (and potential savings) in a Canadian context, there doesn't appear to be peer reviewed research backing up these estimates. Moreover, some data appears to suggest new AT users tend to be former transit users, complicating the calculations involved to determine reduced vehicle kilometers travelled.
Longitudinal research on commute mode and health outcomes over time.	• There is need to track and get information regarding commuting mode and some health outcomes over time.
Research on built environments (including barriers) and PA (including AT) among people living with disabilities.	• There is a need for research examining changes in urban design on PA among people with disabilities.<br>• Again, without lacking specific information, I would say that there is a lack of focus on people who experience disability related to the built environment supporting PA, particularly if their disability does not relate to using a wheelchair.<br>• AT barriers for people with disabilities that include but go beyond mobility impairments. For example, there remains little research in a Canadian context on the experiences of people with invisible disabilities with biking and walking.
Stronger study designs including an emphasis on natural experiment evaluations and large prospective cohort studies to explore effects of changes.	• There are limited prospective studies on how designs can increase PA.<br>• There is a need for research examining changes in built environment features related to cycling infrastructure among residents in the city of Calgary. Does a 1 m distance decrease mortality rates in cyclists?

Research on built environments and injury (e.g., traffic collisions from AT).	• Many researchers have studied the relationship between built environments and injury (by reviewing traffic collisions) but they do not connect these outcomes back to PA or AT. These two areas do not seem to talk to each other. There needs to be a stronger connection between these public health data sources and police reported collisions sources. Ideally this would also be connected back to emergency department data to get information on outcomes.<br>• Complete Streets/Vision Zero/Safe Systems Approach have contributed significantly to the understanding of road users and the built environment. What is less clear and less studied is the link with PA and injury, e.g., a recent study shows increased cycling didn't lead to increased injuries after built environment changes in the short term, but longer term what are the outcomes.<br>• Collisions on city streets involving people walking and biking are notoriously underreported. Collision reports are usually collected by police forces. Reports are often prompted by insurance requirements which incentivizes vehicle reports, leaving a gap in collision data for the most vulnerable road users. Municipalities often rely on collision rates to prioritize intersection and street upgrading projects for safety. The lack of available data about pedestrian and cycling collisions means that these road users are not easily represented in project prioritization and get left out. How do I address this? What research or methods can help?
Need for national data on trip chaining (i.e., a trip that involves multiple purposes/stops), multi-modal travel (using more than 1 more of transport for a trip), and non-work or school AT.	• There is a need for reliable national data, broken down by demographic, on trip chaining, non-work-related transportation and anything outside of the "normal" questions we ask. Rationale: What is currently advanced has been done inside a patriarchal/colonial/capitalist/silo-ed system which is biased towards one bread winner moving to a workplace every weekday and motorized technology. Our census data, for example, is only marginally useful on transportation because it was developed around adult men in a very different work environment. We know next to nothing, on a national basis, about how children move, how their female relatives move, and how they get active when reaching recreation. We know very little about retirees, and know less about Indigenous populations or agricultural workers, or anyone outside of the 9-5. This is due to not having a national household travel survey and is rooted in an overt emphasis on studying car culture.
Use of a systems thinking approach to AT to understand how multiple built environment factors influence behaviour.	• High level, systems analysis-based transport behaviour research, especially research that analyzes collections of factors that work to create a societal-level structure rather than individual components in isolation. Rationale: Most built environment studies focus on one factor. Whereas few studies listing all of the characteristics of a healthy built environment instead and compare them as a form of matrix. We tend to look at one factor in in isolation and look for an answer, but it is rarely there. Built environment factors include cultural factors, for example. Protected bike lane classifications, for example, need to distinguish between those that exist surrounded by residential traffic calming, or designs that allow human beings to ride bicycles side by side while talking to each other. One policy can torpedo otherwise well-meaning initiatives and when we look at them in isolation, we miss most of it.
Evaluation research of built environment interventions (e.g., street retrofitting, changing school boundaries, traffic calming) that promote AT to school and workplaces.	• There is a need for intervention studies that promote active traveling to schools for children and workplace for adults that focus on the built environment.<br>• Benefits of simple administrative methods to encourage AT such as ensuring that students attend the school closest to their residence vs. specialized programs with catchment areas that cover the entire city or larger portions thereof. Many of our schools have catchment areas for which it isn't realistic that many students could participate in active travel - this limits AT and also increases motor vehicle transport and congestion/safety issues near schools.<br>• Retrofitting streets to encourage/support more active travel to school.<br>• There is a lack of research on how changes to the built environment by retrofitting streets through traffic calming affects different types of road users (e.g., cyclists vs drivers). Rationale: While research has shown the effectiveness of traffic calming within the built environment on slowing down vehicle speeds it has not done a strong job at reviewing what types of traffic calming interventions are more conducive to AT users to increase/support PA.<br>• Research linking physical traffic calming measures and observed changes in traffic behaviour to levels of comfort and participation in AT, walking and cycling, to school. We know that there are lots of health and environmental benefits of AT, but it would be good to have a stronger link to traffic calming to justify investments in physical changes to make those modes safer and more attractive.

Multi-sectoral research on built environments and PA.	• Identifying the similarities and differences between engineering research related to built environment and health promotion/behaviour research and where there are similarities, making recommendations on how to bridge these concepts and creating shared language. Rationale: there seems to be similar concepts being researched in both engineering and health academic fields, and because these are happening in separate spheres, and using different terms and frameworks, the knowledge is not being effectively pooled or shared across these sectors. There would be great value in connecting these two fields where there are shared objectives and goals to leverage the expertise of both fields.<br>• Culture-based, communications-based, political research and theories of change that look at the implementation of built environment changes from a social-political perspective rather than a strict urban planning or health lens. We need more studies that look at the "sexiness" of messaging, the impact of that messaging, and how/where it can be used effectively. In a similar vein government-initiated health-based studies tend to provide results that are bureaucratic and, health based, which is not surprising. To get out of the box answers, more collaboration should be done with outsiders, specifically artists, communicators, agitators, architects, biologists, musicians, psychologists. A city and a built environment is an expression of a living being. Research should give this some consideration. Even a "wild-card" team member, coming from a completely different background, should be scored highly. Under this umbrella, although of primary significance, I would add research rooted in Indigenous culture. By this I do not mean "about Indigenous people" but research that takes a different lens and applies it to the research itself - something more holistic, something about connectedness, where the outcomes are different and have a value that is not always assigned to it.
Evaluation of speed limit reduction to support AT and PA.	• I need more research to establish whether lowering posted speed limits will effectively impact driver speeds without also making infrastructure changes. What conditions are necessary to make this an effective change? What enforcement is needed? Rationale: lowering neighbourhood posted speed limits without changing the built environment is an increasingly popular approach in Canadian municipalities. Some research is available but the evidence supporting this as an effective measure is still inconclusive.
Research on built environments and PA (e.g., AT) in rural and non-urban communities.	• There is limited research on how the built environment affects PA in rural communities.<br>• Issues faced by people living rurally and remotely in terms the build environment. Their experience it quite differently than those in urban areas.<br>• Expanding on priority #3, the built environment seems to be associated with various domains of PA depending on different types of communities involved. There is a need to investigate what features of the built environment are important for Canadians at different stages across the lifespan and in different types of communities (i.e., urban, suburban, rural, remote). Rational: In investing the Canadian Active Living Environments (Can-ALE) tool, differences were found by researchers (Colley, 2019) for associations with PA for children verses youths and adults as well as for AT vs. leisure PA. Neighbourhoods with high Can-ALE scores were found to support walking and AT, but not necessarily leisure PA. Moreover, low Can-ALE scored neighbourhoods seemed to be potentially better for children. The results of this study primarily highlight that we are only at the beginning stages of understand the interactions between the built environment and PA.<br>• There is a need for research to understand changes in the built environment to promote AT and PA in smaller communities that have high rates of driving and lower rates of transit use among the general population. Many studies are often in urban settings that limit the ability for practitioners working in smaller communities to apply the findings.<br>• There is a need for research exploring how to increase AT outside of the central neighbourhoods of large, densely populated cities. Particularly, how to encourage suburban residents to use AT to transit, and how to increase AT rates in the many smaller cities and towns in Canada where AT trips are short, but not taken, and rather car trips dominate. Rational: AT is a core domain of PA (Hallal 2012, Prince 2019), but Statistics Canada census data for the trip to work clearly shows that outside of the central areas of large Canadian cities, AT rates are quite low. With significant funds being spent on AT at present, research is needed to understand where to build this infrastructure in order to best drive increased AT rates. Given that most Canadians live outside of central city areas, increases in AT rates are most likely to come from finding the factors that will increase AT in suburbs and smaller towns and cities where AT rates are low. AT is likely to look very different in suburbs and small towns and cities than it looks like in dense downtown neighbourhoods and a separate research literature is likely to be needed.<br>• Barriers to rural AT in Canada. While stakeholders have reported that there is a capacity and knowledge gap between rural and urban municipalities in Canada, there remains little research on the granular AT needs of rural municipalities and more specifically, what kinds of

	resources they want and need. In that vein, there is also a research gap on the impacts of rural municipalities relying on consultants for their AT planning. Research could examine whether there are positive or negative repercussions to relying on consultants compared to urban centres that have AT planners on staff.
Research to understand how to promote AT across all seasons.	• Il est nécessaire de mener des recherches sur comment favoriser la mobilité active et durable en toute saison auprès de la population générale. Justification : Les déplacements actifs, pour se rendre au travail, à l'école ou encore à l'épicerie, sont reconnus comme des piliers essentiels de la mobilité durable et sont une façon simple d'intégrer l'activité physique au quotidien. À titre d'exemple, une étude montréalaise a révélé que les utilisateurs de transports en commun peuvent remplir jusqu'à 25 % des recommandations journalières en matière d'activité physique grâce aux trajets effectués à pied entre le domicile, le réseau de transport et le lieu de travail ou d'étude. Or, la saison hivernale est moins propice à l'utilisation des modes de transports actifs, comme la marche ou le vélo, pour les déplacements du quotidien. Des recherches plus approfondies sur les freins et leviers d'action (aspects motivationnels, enjeux de sécurité, conditions météorologiques etc.) à déconstruire ou au contraire à mobiliser pour promouvoir les déplacements actifs en toute saison seraient pertinentes. / Research is needed on how to promote active and sustainable mobility in all seasons among the general population. Rationale: Active travel to work, school and the grocery store is recognized as a key pillar of sustainable mobility and a simple way to integrate PA into daily life. For example, a Montreal study found that public transit users can meet up to 25% of their daily PA recommendations by walking to and from work or school. However, the winter season is less conducive to the use of active modes of transportation, such as walking or cycling, for daily commuting. Further research on the brakes and levers of action (motivational aspects, safety issues, weather conditions, etc.) to be deconstructed or, on the contrary, mobilized to promote active travel in all seasons would be relevant.
Research examining changes in the urban heat island (built environment) on extreme heat among equity seeking groups and its impact on AT and PA.	• There is a need for research examining changes in the urban heat island (built environment) on extreme heat among equity seeking groups and its impact on AT and PA. Rationale: Cities are creating urban heat island maps that can be used to influence sustainable transportation modes. Municipalities are not yet applying this in this way yet. The equity overlay is especially important because those that are disproportionally impacted by systemic inequities further exacerbated by heat exposure and poor mobility options, need better public policy and planning approaches. The more evidence we have to support this, the more municipalities would be incentivized to act.
Evaluation research on built environment interventions that promote leisure time PA among children and adults.	• There is a need for intervention studies that promote leisure time PA among children and adults that focus on environmental factors.<br>• There is a need for further research on how the physical/built environments influences children's and adults' leisure time PA.<br>• There is a need for evidence-based studies to assist and inform the recreation and leisure industry and their location within the built environment. Few researchers publish with their community partners in j the recreation and leisure industry.
Research to evaluate the benefits of 15-minute neighbourhoods on AT and PA.	• 15-min neighbourhoods have emerged as an important Planning framework to advance public health and climate resiliency. AT and PA are key elements of a 15-min neighbourhood. However, local municipal research is illustrating the important intersection between land use and amenity prevalence with the quality of the pedestrian environment. More academic research in this area would support practical evidence informed evolution of 15-min neighbourhoods and their supporting policy frameworks.
Research exploring the association between investment in maintenance of infrastructure (including cycling and walking paths and green spaces) and PA levels.	• Association between investment in maintenance of infrastructure (including cycling and walking paths and green spaces) with PA levels.<br>• Quantifying demand for AT infrastructure capital and maintenance funding. While there have been studies in Canada that examine content of AT plans (e.g., the TCAT report examining AT plans from an equity lens) there does not appear to be peer reviewed research on financial commitments in municipal AT plans or the extent to which these plans remain unfunded. Moreover, there is a lack of research on funding for AT infrastructure maintenance. As AT networks are expanded, and as AT assets age, maintenance management will become an increasingly complicated task, yet there is little research on this in the Canadian context including barriers to winter maintenance.
Research to better understand what outdoor features of the built environment (e.g., parks, recreation areas, play areas, benches, water	• There is a need to understand what parks, recreation areas, outdoor furniture (play areas, benches, water fountains, bike parking, etc.), and destination mixes will encourage Canadians to be active outside their homes. Rational: Nearly 80% of Canadians report that they have parks and recreation spaces in their neighbourhood (https://health-infobase.canada.ca/pass/); however, we do not know how many people use these facilities, nor do we know what are the factors that make for a successful park or recreation facility. These factors might include distance, access to transit, safety or aspects of the actual features at the parks and recreation facility. Furthermore, in neighbourhoods like the Plateau

fountains, bike parking) encourage Canadians to be active outside their homes.	in Montreal, the Glebe in Ottawa, the Annex in Toronto and other similar neighbourhoods, there is high demand for Canadians to be outside enjoying certain types of shopping, recreation, dining and other mixes of destinations. However, these neighbourhoods are unevenly distributed among Canadians. Given the diverse zoning, features and preferences of Canadian individuals and the neighbourhoods they live in, further research is needed to understand how to design the neighbourhood so that individuals are out and about engaging in reduced sedentary activity. This is particularly important give that many forms of recreation are disproportionately available to Canadians of high incomes. For example, many camping sites or parks are only accessible by car, or recreation areas in cities may have poor transit access.
Research on how greening urban neighbourhoods and impact of national guidance/standards for green spaces can increase PA	• There is limited research on how creating green urban neighborhoods can increase PA.<br>• There is a lack of research on how urban green space can affect PA.<br>• Impact of national guidance/standards for green spaces on local implementation.
Evaluation research on built environment interventions that promote PA in schools and workplaces.	• There is a need for intervention studies that promote PA among children and adults in schools and workplace (respectively) that focus on environmental factors.
Develop valid and reliable measures of active outdoor play and explore relationships with features of the built environment.	• Research is needed to develop valid and reliable outdoor play measurement tools and look at how levels of children's active play vary by features of the built environment, including safety and distance to parks/playgrounds. Rationale: There is a lack of data in this area.
Evaluation of changes in environmental/residential density and PA including AT.	• There is need to understand the potential association of more dense environment and PA as well as fitness levels.<br>• There is a need for further research examining changes in the residential density on the level of PA among the residents. While the relationship between diversity, places of interest and safety was conclusive, the relationship b/w density on level of activity was inconclusive.<br>• There is need for research examining the changes in quality of public realm in a mixed-use or commercial area affect the duration of people hanging out at a certain location and therefore has increased income. A study by RMIT identified that higher density and walkability in neighbourhoods has positive relationship to local income and employment, property values and retail trade.<br>• Relationships between community layout (mix of land uses, roadway layout etc.) and population density with AT or transit use. We're continuing to experience urban sprawl which creates longer trips which may become less practical for AT. Having a clear link would help to advocate for more dense complete communities.
Research on built environments and PA rooted in Indigenous culture.	• Culture-based, communications-based, political research and theories of change that look at the implementation of built environment changes from a social-political perspective rather than a strict urban planning or health lens. We need more studies that look at the "sexiness" of messaging, the impact of that messaging, and how/where it can be used effectively. In a similar vein government-initiated health-based studies tend to provide results that are bureaucratic and, health based, which is not surprising. To get out of the box answers, more collaboration should be done with outsiders, specifically artists, communicators, agitators, architects, biologists, musicians, psychologists. A city and a built environment is an expression of a living being. Research should give this some consideration. Even a "wild-card" team member, coming from a completely different background, should be scored highly. Under this umbrella, although of primary significance, I would add research rooted in Indigenous culture. By this I do not mean "about Indigenous people" but research that takes a different lens and applies it to the research itself - something more holistic, something about connectedness, where the outcomes are different and have a value that is not always assigned to it.
Research to understand the impact and importance of infrastructure (hard interventions) versus	• Understanding the relative impact/importance of infrastructure (or hard interventions) versus behavioural factors (or soft interventions) on AT utilization and mode shift. From some initial literature scans, it seems like there is a strong evidence base looking at the impact of infrastructure development on AT use, and there is also strong expert consensus that infrastructure should be paired with behavioural

behavioural factors (soft interventions) on AT and mode shift.	interventions (e.g., addressing social norms, motivation, perceived safety, skills, etc.) to optimize utilization. However, the relative impact of these two different interventions has not been able to be explored. In some review papers, this gap has been acknowledged and explained by challenges with study design.
Explore changes to workplaces due to the pandemic across marginalized communities (e.g., 2SLGBTQQIA+, racialized, immigrants, etc.).	• There is a need for intervention studies that promote PA among children and adults in schools and workplace (respectively) that focus on environmental factors.
Research on the AT needs of Indigenous communities.	• AT needs of First Nations, Inuit and Metis communities. Research could examine the experience of urban Indigenous people as well as what culturally safe AT facilities could look like on reserve.
Research to evaluate the effectiveness of low-cost and short-term built environment interventions in deprived neighbourhoods.	• Research into effectiveness of low-cost, short term interventions in deprived neighbourhoods.
Research exploring international comparisons on built environments and PA.	• Intercontinental transport research, specifically research that looks at comparison/contrast of locations outside Canada with those within Canada. Rationale: Far too much Canada-only or US only research into built environment and data.<br>• A research priority should be investigating the potential for Canadian best practice communities for PA and the built environment, vs. looking for best practices internationally. Rational: I think it is still an open question whether there is enough variation in Canadian built environments to develop best practices based on higher rates of PA in one community vs. another. What I mean is that in a country with a great diversity of neighbourhood types and city planning, it should be possible to identify exemplar cities and neighbourhoods and promote the built environment features and policies that created those high levels of PA to other communities that wish to replicate those successes. However, Canadian neighbourhoods tend to have very similar features with the exception of older central neighbourhoods of large cities. There is a great deal of homogeneity in new home and neighbourhood construction in Canada in the post war years, such that a suburb in Halifax and in Lethbridge or Kamloops all have a great deal in common. This suggests first that more research should be done to investigate whether differences in PA can be placed with differences in the design of the post-war communities and neighbourhoods. This would be the first stage of the research. My suspicion is that a more fruitful area of investigation will be to look for best practices in community and neighbourhood design in neighbourhoods outside of Canada, and the challenge for policy makers will be to investigate how best to import these design principals in ways that can be applied in a Canadian context. This suggests that international collaborations on research on PA and the built environment and the application of scales such as Can-ALE, Can-BICS and other measures of the built environment will need to increasingly be applied internationally.
Need for higher resolution of traffic-related air pollution for arterials, collectors and local rounds to inform planning.	• Higher resolution of traffic related air pollution for arterials, collector's and local roads. Currently there are a cruder set of setback recommendations that could be refined to better inform planning processes.
Research to explore the connections between AT and outdoor play in children.	• What are the connections between AT and outdoor play and how might these be addressed within national, provincial and local AT strategies currently being developed?
Research using a "neighbour-led" approach to activating neighbourhoods.	• There is a need for research looking at activating neighbourhoods, using a "neighbour-led" approach (i.e., what works to motivate and sustain neighbourhood led initiatives). I can't provide much in the way of rationale as I haven't delved into the literature to that degree.

Need for alternate statistical methods that account for the interaction between multiple built environment factors and PA recognizing that factors may not act in isolation of each other.	• There is a need to conduct innovative secondary statistical analysis on datasets to determine the key factors of the built environment that support PA. Rational: Extensive research has been conducted on features of the built environment that are associated with increased PA. Features such as green canopy, access to parks, sidewalks, destinations, bike lanes, etc. have been found to be associated with increased PA. However, with so many factors available, that statistical analysis drawing upon factor analysis or other tools should be a priority in the short to medium term to determine whether some of these features are more important than others. Potential risks of multicollinearity in model building require that work be done to tighten our understanding of which factors are most important (recognizing that the importance of various factors may change for different groups or settings).
Research examining the availability and condition of sport and recreation infrastructure to support participation in both "organized" (e.g., registered sport activities) and "unorganized" (e.g., casual sport activities, cycling, etc.) sport and recreation.	• There is a need for research examining the availability and condition of sport and recreation infrastructure to support participation in both "organized" (e.g., registered sport activities) and "unorganized" (e.g., casual sport activities, cycling, etc.) sport and recreation. A further evaluation by socio-economic factors (e.g., which neighbourhoods have what infrastructure, and in what condition) would also be helpful. Rationale: There is limited available data on the state of sport and recreation infrastructure in general. The additional evaluation by socio-economic could help identify communities in greatest need of investment.
Research exploring built environments and PA in low-income countries.	• Lack of evidence from low-income countries.
Research on surface (e.g., sealed, natural surfaces, crushed gravel) and path types to promote AT.	• The benefits and drawbacks of sealed paths compared to natural surfaces like crushed gravel in terms of getting more people riding.
Research to identify the most effective types of signage and wayfinding to promote active transport.	• How different types of signage and wayfinding can help people use active transport - what is most effective. In Australia signs tend to be at the height of car-driving signs rather than wayfinding being adapted for people on bikes.
Evaluation of AT infrastructure developed during the COVID-19 pandemic.	• How can we conserve and expand AT routes that have come about during the COVID-19 pandemic?
Natural experiment evaluations of park design on PA and sport.	• Il est nécessaire de mener des recherches sur l'usage efficient des parcs et leur rôle par rapport aux problèmes d'enjeu de santé publique tel que la violence urbaine où l’activité physique sportive serait un levier pour la population. Il y a un manque d’évaluations complète sous forme d’expériences naturelles de l’effet aménagement des parcs sur l'activité physique et sportive pour lutter contre la violence urbaine. / There is a need for research on the efficient use of parks and their role in addressing public health issues such as urban violence where PA is a lever for the population. There is a lack of comprehensive evaluations in the form of natural experiments of the effect of park design on PA and sport to combat urban violence.

Note: some research priorities submitted by respondents may appear in multiple content priorities. AT – active transportation, PA – physical activity
