# Supplementary material for: Prioritizing a research agenda on built environments and physical activity: a twin panel Delphi consensus process with researchers and knowledge users
Source: Int J Behav Nutr Phys Act. 2023 Dec 7;20:144. doi: 10.1186/s12966-023-01533-y (PMC10704660; doi:10.1186/s12966-023-01533-y)
Supplement: Supplementary file 4 — Additional file 4: Table S3. Research priorities in the built environments and physical activity identified by researchers in round 1 and rating and ranking from round 2. [file 12966_2023_1533_MOESM4_ESM.pdf]

Supplementary table 3. Research priorities in the built environments and physical activity identified by researchers in round 1 and rating and ranking from round 2

Round 2 rank	Research priorities	Number of times in top 10	Average rating (higher better)	Average top 10 ranking (lower better)
**1**	Implementation science research on ***how*** to use current research findings to inform policy action to create changes in the built environment. How to move research into practice.	24	4.4	3.8
**2**	Stronger study designs including an emphasis on natural experiment evaluations, randomized controlled trials (RCTs)/quasi-experimental studies and large prospective cohort studies to establish causation.	20	4.0	5.2
**3**	Research to examine the interaction between natural and built environments and climate adaptive approaches (e.g., climate adaptation).	18	4.2	3.9
**4**	Research on inequities in the built environment for PA.	17	4.1	4.2
**5**	Research that incorporates Indigenous (e.g., truth and reconciliation) and climate change perspectives when exploring built environments and PA.	16	3.7	5.9
**6**	Research on land-use policies and how these impact on physical activity.	16	3.5	6.0
**7**	Research to understand how the built environment affects active aging, including social isolation (i.e., research on how to design age-friendly communities, what built environment features support physical activity among older adults).	15	4.0	6.7
**8**	Research to understand how changes to street environments (e.g., slow or complete streets, pedestrian malls, bike lanes) impact on use and safety (e.g., less vehicle travel, reduction in pedestrian and cyclist injuries/fatalities).	15	3.9	5.8
**9**	Research on cost effectiveness evaluations of built environment interventions.	14	4.0	5.6
**10**	Need for participatory processes in research to ensure research is inclusive of a diversity of voices and captures experiential knowledge.	13	3.8	6.1
**11**	Research on features of the built environment that support PA among people living with disability.	12	3.9	5.9
**12**	Research to understand what policy levers are available to promote PA among urban populations.	11	3.7	5.3
**13**	Research that applies a whole of systems approach to built environments and PA.	10	3.7	4.4
**14**	Research investigating how the built environment can enhance or inhibit the effectiveness of PA interventions.	10	3.7	5.5
**15**	Application of a life course perspective when examining built environment as a determinant of PA.	10	3.6	4.2

**16**	Research to examine how structural racism and discrimination contribute to limited PA opportunities/resources across marginalized communities.	10	3.6	6.1
**17**	Residential relocation studies to assess how changes in the built environment are associated with changes in PA.	9	3.5	6.2
**18**	Research on impacts of built environment on PA for different social groups (e.g., women, those with low income, gender diverse, low education, different ethnicities, older adults).	9	4.0	6.6
**19**	Research on built environments that support active outdoor free play in school-aged children.	9	3.6	5.0
**20**	Research to understand travel mode choice and influences on mode choice among different population groups (e.g., women, older adults, specific racial or ethnic groups).	9	3.4	6.6
21	Research to examine the interaction between multiple environmental factors and mediators of the relationship between environments and PA.	9	3.3	5.2
22	Intervention research exploring how to influence AT to school in children.	8	3.5	5.4
23	Mixed methods research (e.g., qualitative and quantitative) to understand what is available and how it is perceived and barriers/facilitators of use.	8	3.5	7.0
24	Standardized methods for measuring elements in the built environment related to context-specific PA.	8	3.4	5.3
25	Longitudinal research combining GIS and GPS to device measures of physical activity to assess location of PA.	8	3.3	6.3
26	Research integrating active transportation and health benefits in traffic models.	7	3.5	7.0
27	Development of a multi-sectoral and multidiscipline research framework for the built environment and PA.	7	3.4	4.4
28	Need for modeling studies into co-benefits of built environment changes beyond PA.	7	3.4	5.0
29	Research on built environments in rural and small-town communities and other urban typologies.	7	3.4	7.0
30	Research on impact of built environments on PA in low- and middle- income countries.	7	3.3	4.6
31	Research examining the interaction between the social and built environments including social norms (e.g., active outdoor play, independent mobility).	6	3.8	7.3
32	Better understanding of perceived vs. objective/actual environments and PA.	6	3.1	5.8
33	Research on built environment interventions for PA across different contexts (e.g., within and outside neighbourhoods, specific locations).	5	3.4	5.6
34	Research that applies behaviour change theories to support behaviour change interventions.	5	3.2	4.2
35	Research to understand what components of the built environment are also associated with sedentary behaviour.	5	3.1	5.6

36	Development of better self-report measurement tools for travel behaviour that account for multi-modal trips or selecting different modes for different purposes or days (e.g., tools that move beyond "in a typical day how do you travel?").	5	3.1	6.0
37	Research to establish threshold values of environmental attributes associated with PA.	5	3.0	4.8
38	Research using international comparisons and a global lens.	5	3.0	9.0
39	Research on built environment interventions in those at risk for poor mental health.	4	3.4	4.5
40	Research on impact of gentrification on PA.	4	3.2	5.5
41	Research to understand what environmental factors are related to different kinds of PA (e.g., bicycle use, walking, running, climbing, playing) compared to physical activity levels in general.	3	3.2	7.3
42	Development of new analytical approaches (e.g., machine learning, latent class modeling, simulation models).	3	3.1	5.0
43	Research to understand how different environments may affect the psychological response to PA and other movement-related behaviours.	3	2.9	6.0
44	Better understanding of to what extent are built environment relationships with overweight and obesity mediated by PA.	3	2.6	9.0
45	Research on built environments that support non-travel related PA.	2	3.3	9.0
46	Research on e-microbilities (e.g., e-scooters, e-bikes) and their interaction with other modes of transportation and impacts on PA.	2	2.9	3.5
47	Research using international comparisons of environmental attributes and PA.	2	2.9	4.5
48	Research into other ways to restructure the environment to support PA in non-traditionally researched settings such as primary care and rehabilitation.	0	2.7	0

AT – active transportation, PA – physical activity
