# Supplementary material for: Prioritizing a research agenda on built environments and physical activity: a twin panel Delphi consensus process with researchers and knowledge users
Source: Int J Behav Nutr Phys Act. 2023 Dec 7;20:144. doi: 10.1186/s12966-023-01533-y (PMC10704660; doi:10.1186/s12966-023-01533-y)
Supplement: Supplementary file 5 — Additional file 5: Table S4. Research priorities in the built environments and physical activity identified by knowledge users in round 1 and rating and ranking from round 2. [file 12966_2023_1533_MOESM5_ESM.pdf]

Supplementary table 4. Research priorities in the built environments and physical activity identified by knowledge users in round 1 and rating and ranking from round 2

Round 2 rank	Research priorities	Number of times in top 10	Average rating (higher better)	Average top 10 ranking (lower better)
**1**	Research to better understand current inequalities in access to built environments and how changes in built environments can reduce social inequalities.	14	4.2	5.4
**2**	Research on impacts of built environments on physical activity for different social groups (e.g., women, very young children, adolescents, older adults, those with low income, homeless, gender diverse, occupations, low education, different ethnicities, 2SLGBTQQIA+)	12	4.2	4.1
**3**	Research exploring economic evaluations of AT infrastructure that promotes physical activity.	12	4.1	5.1
**4**	Research that explores the interaction between climate change and built environments and effects on PA.	12	4.0	5.3
**5**	Longitudinal research on commute mode and health outcomes over time.	12	3.8	5.8
**6**	Research on built environments (including barriers) and PA (including AT) among people living with disabilities.	11	4.2	6.0
**7**	Stronger study designs including an emphasis on natural experiment evaluations and large prospective cohort studies to explore effects of changes.	11	3.8	5.9
**8**	Research on built environments and injury (e.g., traffic collisions from AT).	9	3.9	5.1
**9**	Need for national data on trip chaining (i.e., a trip that involves multiple purposes/stops), multi-modal travel (using more than 1 more of transport for a trip), and non-work or school AT.	9	3.8	6.4
**10**	Use of a systems thinking approach to AT to understand how multiple built environment factors influence behaviour.	8	3.9	4.5
**11**	Evaluation research of built environment interventions (e.g., street retrofitting, changing school boundaries, traffic calming) that promote AT to school and workplaces.	7	3.9	6.4
**12**	Multi-sectoral research on built environments and PA.	7	3.8	7.7
**13**	Evaluation of speed limit reduction to support AT and PA.	7	3.7	5.3
**14**	Research on built environments and PA (e.g., AT) in rural and non-urban communities.	6	3.7	5.5
**15**	Research to understand how to promote AT across all seasons.	6	3.6	8.0
**16**	Research examining changes in the urban heat island (built environment) on extreme heat among equity seeking groups and its impact on AT and PA.	6	3.3	4.0

**17**	Evaluation research on built environment interventions that promote leisure time PA among children and adults.	6	3.2	4.5
**18**	Research to evaluate the benefits of 15-minute neighbourhoods on AT and PA.	5	3.9	4.6
**19**	Research exploring the association between investment in **maintenance** of infrastructure (including cycling and walking paths and green spaces) and PA levels.	5	3.9	5.4
**20**	Research to better understand what outdoor features of the built environment (e.g., parks, recreation areas, play areas, benches, water fountains, bike parking) encourage Canadians to be active outside their homes.	5	3.5	4.6
21	Research on how greening urban neighbourhoods and national guidance/standards for green spaces can impact PA.	5	3.5	5.6
22	Evaluation research on built environment interventions that promote PA in schools and workplaces.	5	3.5	6.4
23	Develop valid and reliable measures of active outdoor play and explore relationships with features of the built environment.	5	3.4	2.2
24	Evaluation of changes in environmental/residential density and PA including AT.	5	3.4	4.2
25	Research on built environments and physical activity rooted in Indigenous culture.	5	3.4	6.6
26	Research to understand the impact and importance of infrastructure (hard interventions) versus behavioural factors (soft interventions) on AT and mode shift.	5	3.3	2.6
27	Explore changes to workplaces due to the pandemic across marginalized communities (e.g., 2SLGBTQQIA+, racialized, immigrants, etc.).	5	3.0	6.0
28	Research on the AT needs of Indigenous communities.	4	3.7	5.8
29	Research to evaluate the effectiveness of low-cost and short-term built environment interventions in deprived neighbourhoods.	4	3.5	5.0
30	Research exploring international comparisons on built environments and PA.	4	3.3	7.0
31	Need for higher resolution of traffic-related air pollution for arterials, collectors and local rounds to inform planning.	4	3.1	6.8
32	Research to explore the connections between AT and outdoor play in children.	3	3.2	5.0
33	Research using a "neighbour-led" approach to activating neighbourhoods.	3	3.1	7.7
34	Need for alternate statistical methods that account for the interaction between multiple built environment factors and PA recognizing that factors may not act in isolation of each other.	3	3.0	7.3
35	Research examining the availability and condition of sport and recreation infrastructure to support participation in both "organized" (e.g., registered sport activities) and "unorganized" (e.g., casual sport activities, cycling, etc.) sport and recreation.	3	2.8	8.3
36	Research exploring built environments and PA in low-income countries.	3	2.5	4.3

37	Research on surface (e.g., sealed, natural surfaces, crushed gravel) and path types to promote AT.	2	2.6	9.5
38	Research to identify the most effective types of signage and wayfinding to promote AT.	1	3.4	4.0
39	Evaluation of AT infrastructure developed during the COVID-19 pandemic.	1	3.2	3.0
40	Natural experiment evaluations of park design on PA and sport.	0	2.7	0

AT – active transportation, PA – physical activity
