# Supplementary material for: Prioritizing a research agenda on built environments and physical activity: a twin panel Delphi consensus process with researchers and knowledge users
Source: Int J Behav Nutr Phys Act. 2023 Dec 7;20:144. doi: 10.1186/s12966-023-01533-y (PMC10704660; doi:10.1186/s12966-023-01533-y)
Supplement: Supplementary file 6 — Additional file 6: Table S5. Average ranking of top 20 list in round 3 among researchers reported in order provided to researchers in round 3. [file 12966_2023_1533_MOESM6_ESM.pdf]

Supplementary table 5. Average ranking of top 20 list in round 3 among researchers reported in order provided to researchers in round 3

Item # (as per round 3 list)	Research priorities	Average ranking (1 = most important, 20 = least important)
**1**	Implementation science research on how to use current research findings to inform policy action to create changes in the built environment. How to move research into practice.	1.42
**2**	Stronger study designs including an emphasis on natural experiment evaluations, randomized controlled trials (RCTs)/quasi-experimental studies and large prospective cohort studies to establish causation.	2.26
**3**	Research to examine the interaction between natural and built environments and climate adaptive approaches.	3.97
**4**	Research on inequities in the built environment for PA.	4.66
**5**	Research that incorporates Indigenous (e.g., truth and reconciliation) and climate change perspectives when exploring built environments and PA.	6.76
**6**	Research on land-use policies and how these impact on PA.	6.42
**7**	Research to understand how the built environment affects active aging, including social isolation (i.e., research on how to design age-friendly communities, what built environment features support PA among older adults).	7.42
**8**	Research to understand how changes to street environments (e.g., slow or complete streets, pedestrian malls, bike lanes) impact on use and safety (e.g., less vehicle travel, reduction in pedestrian and cyclist injuries/fatalities).	8.18
**9**	Research on cost effectiveness evaluations of built environment interventions.	9.16
**10**	Need for participatory processes in research to ensure research is inclusive of a diversity of voices and captures experiential knowledge.	10.39
11	Research on features of the built environment that support PA among people living with disability.	11.61
12	Research to understand what policy levers are available to promote PA among urban populations.	11.61
13	Research that applies a whole of systems approach to built environments and PA.	12.16
14	Research investigating how the built environment can enhance or inhibit the effectiveness of PA interventions.	13.66
15	Application of a life course perspective when examining built environment as a determinant of PA.	14.29
16	Research to examine how structural racism and discrimination contribute to limited PA opportunities/resources across marginalized communities.	16.00

17	Residential relocation studies to assess how changes in the built environment are associated with changes in PA.	16.61
18	Research on impacts of built environment on PA for different social groups (e.g., women, those with low income, gender diverse, low education, different ethnicities, older adults).	16.45
19	Research on built environments that support active outdoor free play in school-aged children.	17.74
20	Research to understand travel mode choice and influences on mode choice among different population groups (e.g., women, older adults, specific racial or ethnic groups).	19.24

PA – physical activity
