# Supplementary material for: Prioritizing a research agenda on built environments and physical activity: a twin panel Delphi consensus process with researchers and knowledge users
Source: Int J Behav Nutr Phys Act. 2023 Dec 7;20:144. doi: 10.1186/s12966-023-01533-y (PMC10704660; doi:10.1186/s12966-023-01533-y)
Supplement: Supplementary file 7 — Additional file 7: Table S6. Average ranking of top 20 list in round 3 among knowledge users reported in order provided to knowledge users in round 3. [file 12966_2023_1533_MOESM7_ESM.pdf]

Supplementary table 6. Average ranking of top 20 list in round 3 among knowledge users reported in order provided to knowledge users in round 3

**Item # (as per round 3 list)**	**Research Priorities**	**Average ranking (1 = most important, 20 = least important)**
**1**	Research to better understand current inequalities in access to built environments and how changes in built environments can reduce social inequalities.	1.61
**2**	Research on impacts of built environments on PA for different social groups (e.g., women, very young children, adolescents, older adults, those with low income, homeless, gender diverse, occupations, low education, different ethnicities, 2SLGBTQQIA+)	2.91
**3**	Research exploring economic evaluations of AT infrastructure that promotes PA.	4.35
**4**	Research that explores the interaction between climate change and built environments and effects on PA.	4.48
**5**	Longitudinal research on commute mode and health outcomes over time.	6.30
**6**	Research on built environments (including barriers) and PA (including active transportation) among people living with disabilities.	6.65
**7**	Stronger study designs including an emphasis on natural experiment evaluations and large prospective cohort studies to explore effects of changes.	7.87
**8**	Research on built environments and injury (e.g., traffic collisions from AT).	8.87
**9**	Need for national data on trip chaining (i.e., a trip that involves multiple purposes/stops), multi-modal travel (using more than 1 more of transport for a trip), and non-work or school AT.	9.07
**10**	Use of a systems thinking approach to AT to understand how multiple built environment factors influence behaviour.	10.04
11	Evaluation research of built environment interventions (e.g., street retrofitting, changing school boundaries, traffic calming) that promote AT to school and workplaces.	11.26
12	Multi-sectoral research on built environments and physical activity.	12.04
13	Evaluation of speed limit reduction to support AT and PA.	13.78
14	Research on built environments and PA (e.g., AT) in rural and non-urban communities.	13.65
15	Research to understand how to promote AT across all seasons.	13.87
16	Research examining changes in the urban heat island (built environment) on extreme heat among equity seeking groups and its impact on AT and PA.	15.65

17	Evaluation research on built environment interventions that promote leisure time PA among children and adults.	16.57
18	Research to evaluate the benefits of 15-minute neighbourhoods on AT and PA.	15.52
19	Research exploring the association between investment in **maintenance** of infrastructure (including cycling and walking paths and green spaces) and PA levels.	17.48
20	Research to better understand what outdoor features of the built environment (e.g., parks, recreation areas, play areas, benches, water fountains, bike parking) encourage Canadians to be active outside their homes.	18.00

AT – active transportation, PA – physical activity
